# Shared genetic architecture between neuroticism, coronary artery disease and cardiovascular risk factors

**DOI:** 10.1038/s41398-021-01466-9

**Published:** 2021-06-17

**Authors:** Kristin Torgersen, Shahram Bahrami, Oleksandr Frei, Alexey Shadrin, Kevin S. O’ Connell, Olav B. Smeland, John Munkhaugen, Srdjan Djurovic, Toril Dammen, Ole A. Andreassen

**Affiliations:** 1 Department of Behavioral Medicine and Faculty of Medicine, University of Oslo, Oslo, Norway; 2 NORMENT: Norwegian Centre for Mental Disorders Research, University of Oslo and Oslo University Hospital, Oslo, Norway; 3 Department of Informatics, Center for Bioinformatics, University of Oslo, Oslo, Norway; 4 Department of Medicine, Drammen Hospital, Drammen, Norway; 5 Department of Medical Genetics, Oslo University Hospital, Oslo, Norway; 6 Department of Clinical Science, University of Bergen, Bergen, Norway

**Keywords:** Psychiatric disorders, Biomarkers

## Abstract

Neuroticism is associated with poor health, cardiovascular disease (CVD) risk factors and coronary artery disease (CAD). The conditional/conjunctional false discovery rate method (cond/conjFDR) was applied to genome wide association study (GWAS) summary statistics on neuroticism (*n* = 432,109), CAD (*n* = 184,305) and 12 CVD risk factors (*n* = 188,577–339,224) to investigate genetic overlap between neuroticism and CAD and CVD risk factors. CondFDR analyses identified 729 genomic loci associated with neuroticism after conditioning on CAD and CVD risk factors. The conjFDR analyses revealed 345 loci jointly associated with neuroticism and CAD (*n* = 30), body mass index (BMI) (*n* = 96) or another CVD risk factor (*n* = 1–60). Several loci were jointly associated with neuroticism and multiple CVD risk factors. Seventeen of the shared loci with CAD and 61 of the shared loci with BMI are novel for neuroticism. 21 of 30 (70%) neuroticism risk alleles were associated with higher CAD risk. Functional analyses of the genes mapped to the shared loci implicated cell division, nuclear receptor, elastic fiber formation as well as starch and sucrose metabolism pathways. Our results indicate polygenic overlap between neuroticism and CAD and CVD risk factors, suggesting that genetic factors may partly cause the comorbidity. This gives new insight into the shared molecular genetic basis of these conditions.

## Introduction

Neuroticism is a personality trait that involves the tendency to experience negative emotions^1^, and is associated with psychiatric illnesses such as depression and anxiety disorders^2^. There is growing evidence that neuroticism is also associated with cardiovascular disease (CVD), and CVD risk factors such as high body mass index (BMI)^3^, type 2 diabetes (T2D) and hypertension^4^. Further, some prospective clinical and epidemiological studies indicate that neuroticism increases the risk of coronary artery disease (CAD) and mortality compared to the general population^5,6^. However, the findings are inconsistent and the association is not clearly established^6–9^.

The mechanisms underlying the associations between neuroticism and CVD risk factors and CAD are not known. Neuroticism may contribute to CAD through behavioral mechanisms such as poor health-related behaviors (smoking, sedentary life style, and unhealthy diet) and low adherence to medication and rehabilitation^10,11^. Different biological pathways have also been proposed to explain the higher incidence of CAD in people with neuroticism; dysregulation of the hypothalamic-pituitary-adrenal axis results in increased cortisol levels due to stress, leading to higher daytime cortisol levels which in turn elevates blood pressure, autonomic dysregulation, subclinical inflammation and oxidative stress, while also reducing the number of stem cells^11^. Further, it has been hypothesized that the association between neuroticism and CAD, and its related risk factors is partly caused by genetic pleiotropy between neuroticism and CAD, hypertension, and higher BMI^6–9^.

Twin and adoption studies suggest that heritability accounts for between a third and a half of individual differences in neuroticism^12^. In adolescence and early adulthood, 50–60% of the variance in neuroticism scores is estimated to be attributable to genetic factors^13^. A recent GWAS meta-analysis of neuroticism, with a total number of 449,484 participants, identified 136 independent genome-wide significant loci implicating 599 genes^14^, and underscored the polygenic architecture of this trait.

CAD is also highly heritable, with estimates of 40–50% from family studies^15^. Twin studies found the heritability of CAD to be 55% after controlling for smoking and BMI^16^. GWAS have identified 161 loci associated with CAD^17^. Recent studies, applying Linkage disequilibrium score regression (LDSR), have shown significant positive genetic correlations between neuroticism and CVD risk factors and polygene risk score (PRS) analyses provide further evidence of genetic overlap^18^. Gale et al. showed that PRS for CAD and cigarette smoking, a known CVD risk factor, were positively associated with neuroticism, while PRS for BMI was associated in a negative direction^1^. However, studies based on PRS and LDSR are not able to identify specific genetic loci involved.

Recently developed methodologies are able to identify overlapping genetic loci between two traits beyond genetic correlation^19^. We here apply the conditional false discovery rate (condFDR) analytical approach to a large neuroticism GWAS, to evaluate the polygenic overlap with CAD and 12 CVD risk factors. Further, a large part of the polygenic architecture of neuroticism remains unexplained. Thus, we also leveraged the genetic overlap between neuroticism, CAD, and CVD risk factors to boost the power to discover genetic variants associated with neuroticism conditioned on the genetic effects in associated traits^20–22^.

We analyzed summary statistics from GWAS of neuroticism (*n* = 432,109)^14^, CAD^20^, and 12 CVD risk factors; BMI^22^, WHR^21^, high density lipoprotein (HDL)^23^, low density lipoprotein (LDL)^23^, triglycerides (TG)^23^, total cholesterol (TC)^23^, T2D^24^, c-reactive protein (CRP)^25^, systolic blood pressure (SBP)^26^, diastolic blood pressure (DBP)^26^, pulse pressure (PP)^26^, and cigarettes smoked per day (CIGPRDAY)^27^.

## Materials and methods

### Participants

In the present study, GWAS summary statistics data on neuroticism were available for 432,109 individuals (372,903 individuals from the UK Biobank^28^ and 59,206 individuals from 23andMe, Inc^29^.) who completed a questionnaire on neuroticism and provided DNA for genome-wide genotyping^14^. We meta-analysed the two GWAS summary statistics using METAL^30^.

Between 2006 and 2010, 502,655 community-dwelling people aged between 37 and 73 years and living in the United Kingdom were recruited to the UK Biobank study and completed the baseline survey (http://www.ukbiobank.ac.uk)^28^. They underwent assessments of cognitive and physical functions, mood and personality. They provided blood, urine, and saliva samples for future analysis, completed questionnaires about their social backgrounds and lifestyle and agreed that their information could be used in research.

The 23andMe sample was based on self-reported information from more than 1,000,000 individuals (90% participating in research), through a direct-to-consumer online genetic-testing service since 2006^29^. Participants provided informed consent and participated in the research online, under a protocol approved by the external AAHRPP-accredited IRB, Ethical & Independent Review Services (E&I Review).

### Neuroticism assessment

UK Biobank participants completed the Neuroticism scale of the Eysenck Personality Questionnaire-Revised (EPQ-R) Short Form (12 item)^31^. This scale has been validated in older people against two of the most used measures of neuroticism, taken from the International Personality Item Pool (IPIP) and correlated −0.84 with the IPIP-Emotional Stability scale and 0.85 with the NEO-Five Factor Inventory (NEO-FFI)^32^.

### GWAS summary statistics for CAD and CVD Risk factors

We obtained GWAS summary statistics for CAD (*n* = 184,305)^20^ and the related risk factors for CVD including BMI^22^, WHR^21^, HDL^23^, LDL^23^, TG^23^, TC^23^, T2D^24^, CRP^25^, SBP^26^, DBP^26^, PP^26^, and CIGPRDAY^27^ (*n* = 188,577–339,224 depending on the CVD risk factor). More information on the characteristics of the study samples and inclusion criteria for the different GWAS is given in Supplementary Table 15, and the original publications were also the extensive quality control procedures are described in detail^14,20–24,26,27^. GWAS participants were predominantly of European ancestry, except for SBP, DBP, and PP. There was no sample overlap between participants in the neuroticism sample and those in the CAD or CVD risk factor samples.

### Ethics

All GWAS used in the present study were approved by the local ethics committees, and all the participants gave their informed consent^14,20–24,26,27^. UK Biobank received ethical approval from the Research Ethics Committee (REC reference 11/NW/0382). The current protocol was assessed by Regional Committees for Medical Research Ethics - South East Norway, and no additional institutional review board approval was necessary because no individual data were used. For more details, see Supplementary Methods and the original publications.

### Statistical analyses

To estimate SNP-based genetic correlations between neuroticism, CAD, and CVD risk factors, we used linkage disequilibrium (LD) score regression^33^. The analysis was performed using the Python-based package available at (https://github.com/bulik/ldsc), with the procedure described in the documentation for the package (https://github.com/bulik/ldsc/wiki/Heritability-and-Genetic-Correlation).

We constructed conditional quantile–quantile (Q–Q) plots to visualize cross-trait enrichment^34^. The conditional Q–Q plots compare the association with one trait (e.g., neuroticism) within SNPs strata determined by significant association with a secondary trait (e.g., CAD). Cross-trait enrichment exists if the proportion of SNPs associated with a phenotype increases as a function of the strength of the association with a secondary phenotype, and is shown by a successively leftward deflection from the null line on the conditional Q–Q plot. This can be directly interpreted in terms of the true discovery rate (1-FDR)^35–37^.

To improve the discovery of genetic variants associated with neuroticism, CAD and CVD risk factors we used a condFDR statistical framework^38^. This statistical method is an extension of the standard FDR, and uses genetic association summary statistics from the primary trait of interest (neuroticism) together with those of a conditional trait (e.g., CAD). CondFDR re-ranks the test-statistics of a primary phenotype based on a conditional variable, here the strength of the association with CAD and CVD risk factors. By leveraging the condFDR we increased power and incorporated useful information from a second trait into the analysis, identifying the SNPs more likely to replicate. Altering the roles of primary and secondary phenotypes gives the inverse condFDR value. P-values were corrected for inflation using a genomic inflation control procedure^35^.

We also applied the conjFDR method^35^, an extension of the condFDR, to detect loci showing strong evidence of association with both neuroticism and the given secondary trait. The conjFDR method is defined by the maximum of the two condFDR values for a specific SNP, and estimates the posterior probability for a SNP being null for either trait or both at the same time, given that the *P* values for both phenotypes are equal to, or smaller, than the *P*-values for each trait individually.

We applied a condFDR level of 0.01 and a conjFDR of 0.05 per pairwise comparison. Manhattan plots were constructed based on the ranking of the conjFDR to show the shared genetic risk loci. All SNPs without pruning are shown, and the independent significant lead SNPs are encircled in black. SNPs in the major extended histocompatibility complex and 8p23.1 region were excluded. For more details, see the original^35^ and subsequent publications^39–41^.

### Genomic loci definition

We used FUMA to define the independent genomic loci^42^. SNPs with condFDR < 0.01 and conjFDR < 0.05 were identified as independent significant SNPs, and independent from each other at *r*^2^ < 0.6. Lead SNPs were selected in approximate linkage equilibrium with each other at *r*^2^ < 0.1. To identify distinct genomic loci, all physically overlapping lead SNPs were merged (LD blocks <250 kb apart). The borders of the genomic loci were determined by identifying all SNPs in linkage disequilibrium (LD) (*r*^2^ ≧ 0.6) with one of the independent significant SNPs in the locus. The part of the gene containing all of these candidate SNPs was evaluated as a single independent genomic locus. However, due to the inability to identify the causal variants from GWAS, we cannot rule out that different tag SNPs can represent the same causal locus. The 1000 Genomes Project reference panel^43^ was used to calculate the LD information. The directional effects of the loci shared between neuroticism and cardiovascular traits were assessed by comparing their *z*-scores and odds ratios.

### Functional annotation

We annotated all lead SNPs in condFDR < 0.01, conjFDR < 0.05, and all candidate SNPs in the genomic loci with a conjFDR value < 0.1 having an LD *r*^2^ ≧ 0.6 with one of the independent significant SNPs by using FUMA^42^. We applied another tool to predict the deleteriousness of SNPs on the proteins structure and function; *Combined Annotation Dependent Depletion* (CADD)^44^. Further, we leveraged *RegulomeDB*^45^, a method to predict regulatory functions, and then *chromatin states*, which predict transcription/regulatory effects of chromatin states at the SNP locus^46,47^. We identified loci overlapping with previously reported GWAS associations in the NHGRI-EBI catalog^48^. We also used FUMA^42^ for gene-set enrichment for the genes nearest the identified shared loci represented by Gene Ontology (GO)^49^. The genotype expression (GTEx) resource^50^ was applied to evaluate expression quantitative trait locus (eQTL) functionality of likely regulatory lead SNPs. We corrected all analyses for multiple comparisons.

## Results

### Genetic correlations

Using genome-wide LD score regression analyses, we found non-significant negative genetic correlation (*r*_g_) between neuroticism and BMI (*r*_g_ = −0.0174 (SE 0.0213), *P* = 0.413) and HDL (*r*_g_ = −0.0216 (SE 0.0244), *P* = 0.3765) and positive genetic correlation with neuroticism that was nominally significant for CAD (*r*_g_ = 0.0919 (SE 0.0289), *P* = 0.0015), TG (*r*_g_ = 0.0367 (SE 0.0182), P = 0.0432), WHR (*r*_g_ = 0.065 (SE 0.0269), *P* = 0.0159), and non-significant for DBP (*r*_g_ = 0.0333 (SE 0.0272), *P* = 0.2209), SBP (*r*_g_ = 0.0426 (SE 0.025), *P* = 0.0893), CRP (*r*_g_ = 0.0313 (SE 0.0273), *P* = 0.2526), CIGPRDAY (*r*_g_ = 0.0445 (SE 0.0572), *P* = 0.4371), T2D (*r*_g_ = 0.0377 (SE 0.0337), *P* = 0.2638), LDL (*r*_g_ = 0.0308 (SE 0.026), *P* = 0.2351), and TC (*r*_g_ = 0.0333 (SE 0.0256), *P* = 0.1936) (Suppl. Fig. 1).

### Polygenic overlap

To visually determine the presence of polygenic enrichment across traits, which is a measure of polygenic overlap, we generated conditional Q–Q plots for neuroticism conditioned on CAD and CVD risk factors, excluding CIGPRDAY. Leftward deflection from the null-line in the conditional Q–Q plots reflects polygenic enrichment. The strongest enrichment was observed for neuroticism after conditioning on CAD or BMI, and vice versa (Figs. 1 and 2). There were weaker signs of enrichment in the other traits (Suppl. Figs. 2–21).

**Fig. 1:**
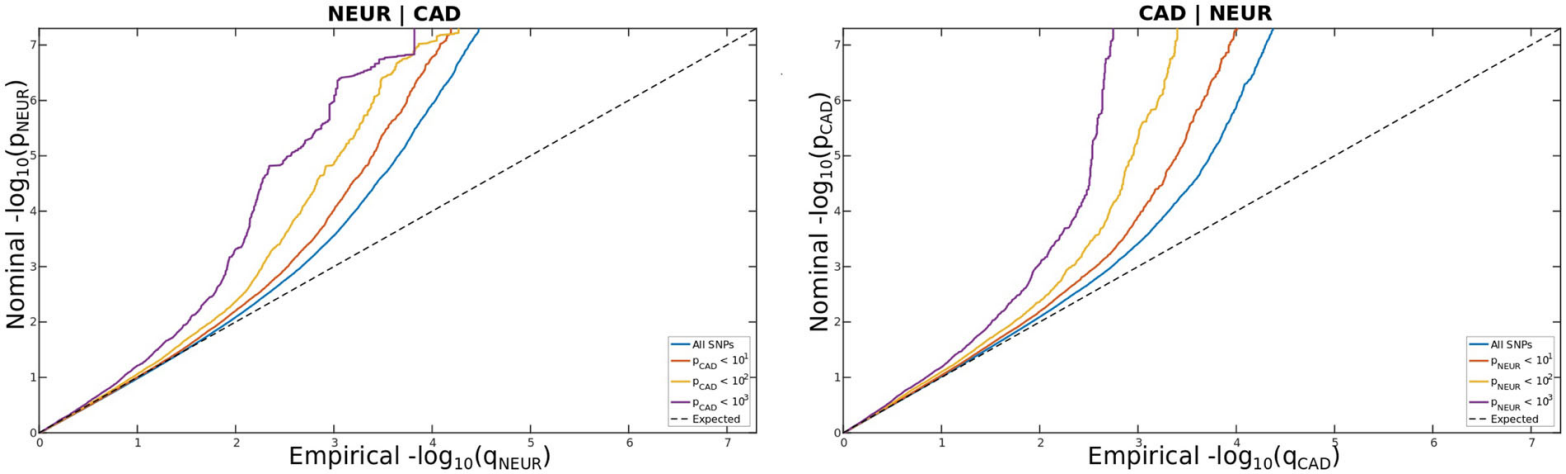
Polygenic overlap between neuroticism (NEUR) conditioned on CAD (NEUR∣CAD) and CAD conditioned on NEUR (CAD∣NEUR). Conditional q–q plots of nominal versus empirical –log 10p values (corrected for inflation) in primary trait (NEUR or CAD) below the standard GWAS threshold of *P* < 5 × 10^–8^ as a function of significance of association with secondary trait (CAD or NEUR) at the level of *P* < 0.1, *P* < 0.01, and *P* < 0.001, respectively. The blue line indicates all SNPs. The dashed line indicate the null hypothesis.

**Fig. 2:**
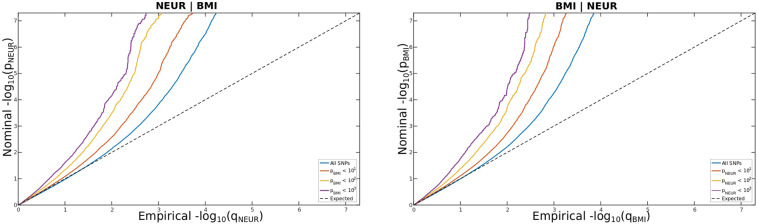
Polygenic overlap between neuroticism (NEUR) conditioned on BMI (NEUR∣BMI) and BMI conditioned on NEUR (BMI∣NEUR). Conditional q–q plots of nominal versus empirical –log 10p values (corrected for inflation) in primary trait (NEUR or BMI) below the standard GWAS threshold of *p* < 5 × 10^–8^ as a function of significance of association with secondary trait (BMI or NEUR) at the level of *p* < 0.1, *p* < 0.01, and *p* < 0.001, respectively. The blue line indicates all SNPs. The dashed line indicate the null hypothesis.

### Shared genetic loci

#### CondFDR

When combining the condFDR analyses for neuroticism and all of the secondary traits, we identify 729 unique SNPs associated with neuroticism conditional on a secondary trait (condFDR < 0.01). A large number of neuroticism SNPs were associated with multiple secondary traits, illustrated by a total of *n* = 1682 significant associations. We identified 154 loci associated with neuroticism conditional on CAD, 140 on BMI, 154 on DBP, 170 on SBP, 102 on WHR and 98 on HDL (Suppl. Tables 2–7). The reverse condFDR analyses identified 122, 344, 140, 264, 102, and 193 loci associated with CAD, BMI, DBP, SBP, WHR, and HDL, respectively, conditional on neuroticism. (Suppl. Tables 2–7). We also identified neuroticism loci conditional on TC, TG, T2D, LDL, CRP, PP, and visa-versa (Suppl. Tables 8–13).

#### ConjFDR

**Fig. 3:**
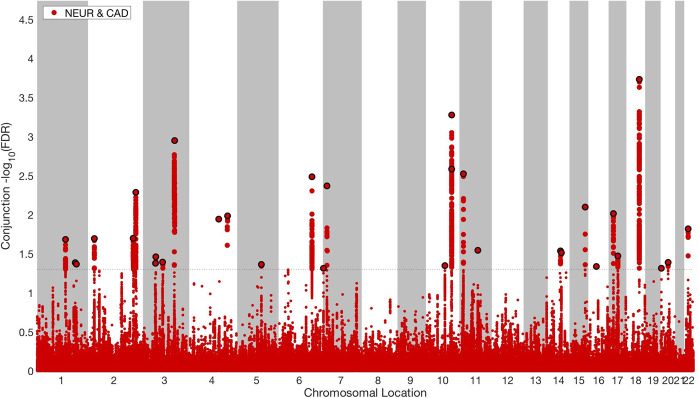
Common genetic variants jointly associated with neuroticism (*n* = 432,109) and CAD (*n* = 184,305) at conjunctional false discovery rate (conjFDR) < 0.05. Manhattan plot showing the –log10 transformed conjFDR values for each SNP on the *y* axis and the chromosomal positions along the *x* axis. The dotted horizontal line represents the threshold for significant shared associations (conjFDR < 0.05, i.e., −log10(conjFDR) > 2.0). Independent lead SNPs are encircled in black. The significant shared signal in the major histocompatibility complex region (chr6:25119106–33854733) is represented by one independent lead SNP. Further details are available in Supplementary Table 2.

**Fig. 4:**
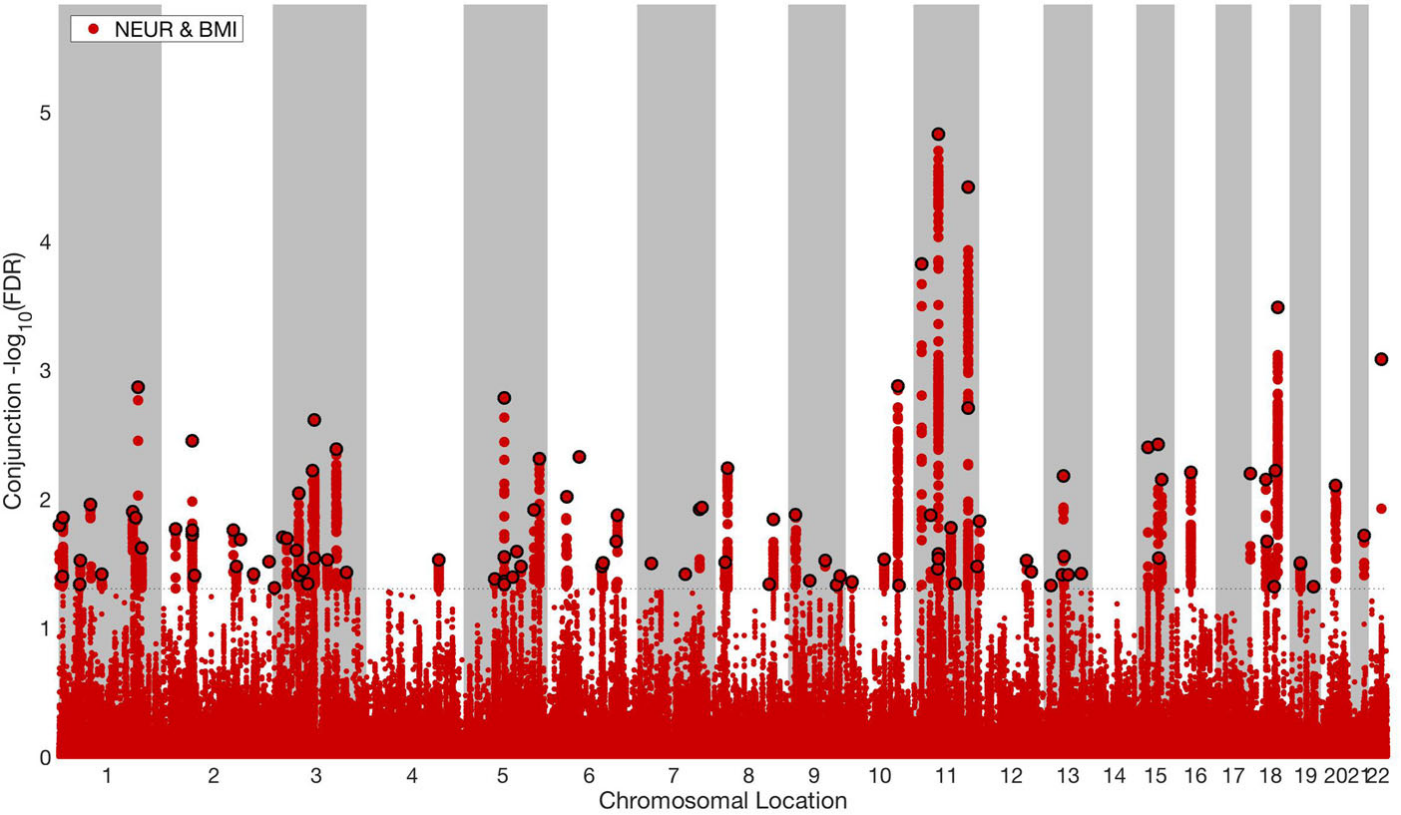
Common genetic variants jointly associated with neuroticism (*n* = 432,109) and BMI (*n* = 184,305) at conjunctional false discovery rate (conjFDR) < 0.05. Manhattan plot showing the –log10 transformed conjFDR values for each SNP on the *y* axis and the chromosomal positions along the *x* axis. The dotted horizontal line represents the threshold for significant shared associations (conjFDR < 0.05, i.e., −log10(conjFDR) > 2.0). Independent lead SNPs are encircled in black. The significant shared signal in the major histocompatibility complex region (chr6:25119106–33854733) is represented by one independent lead SNP. Further details are available in Supplementary Table 3.

To identify the genetic loci jointly associated with both neuroticism and CVD risk factors and CAD, we used the conjFDR method. We identified a total of 345 unique SNPs with significant (conjFDR < 0.05) effects in both traits. A total of 30 distinct genomic loci were jointly associated with neuroticism and CAD (Fig. 3 and Suppl. Table 2). Seventeen of these loci were not identified in the original neuroticism GWAS^14^ and ten were not reported in the original CAD GWAS^20^. Five of the loci are novel in both phenotypes. Ninety-six distinct genomic loci were associated with both neuroticism and BMI (Fig. 4 and Suppl. Table 3); 61 of these loci were not identified in the original neuroticism GWAS^14^ and 17 are novel for BMI. Thirteen were novel in both traits. Moreover, 46 loci were jointly identified between neuroticism and DBP (Suppl. Fig. 22 and Suppl. Table 4). Twenty-nine of these were not previously identified for neuroticism, and 19 were not identified previously for DBP. Seventeen loci are novel for both phenotypes. Sixty loci were jointly associated with neuroticism and SBP (Suppl. Fig. 23 and Suppl. Table 5). Of these loci, 40 were not previously reported for neuroticism. Nine were not previously reported for SBP, and nine are novel for both neuroticism and SBP. We also identified 22 distinct loci shared between neuroticism and WHR (Suppl. Fig. 24 and Suppl. Table 6). Thirteen of these were not identified in the original neuroticism GWAS^14^ and 15 had not been identified in the original WHR GWAS^21^, yielding a total number of eight novel neuroticism risk loci among the shared loci. In addition, 29 distinct genomic loci were associated with both neuroticism and HDL (Suppl. Fig. 25 and Suppl. Table 7); 15 of these loci were not identified in the original neuroticism GWAS^14^, 20 of the 29 loci were novel for HDL, and 11 were novel in both traits.

One locus was shared between neuroticism, CAD, BMI, WHR, and HDL (Table 1 and Suppl. Table 14). The nearest gene for this locus is the pseudogene *RPS3A49*. Several loci were shared between neuroticism and more than one secondary phenotype (Table 1 and Suppl. Table 14). We also identified loci jointly associated (conjFDR < 0.05) with neuroticism and TC (*n* = 17), TG (*n* = 16), T2D (*n* = 15), CRP (*n* = 10), LDL (*n* = 10), PP (*n* = 36), and CIGPRDAY (*n* = 1), respectively (Suppl. Figs. 26–232, Suppl. Tables 8–13, and 15). We visualized the distribution of the shared variants by conjFDR Manhattan plots, where all SNPs without pruning are shown, and the independent significant lead SNPs are encircled in black (Figs. 3 and 4 and Suppl. Figs. 22–32).

**Table 1:** Loci shared between neuroticism and more than one secondary phenotype.

Phenotype	CHR	LEAD SNP	MinBP	MaxBP
BMI, TG, WHR, SBP AND PP	1	rs1460940	72628347	72959392
SBP, DBP, BMI AND PP	2	rs736699	26911509	26932796
SBP AND PP	2	rs343968	44905806	45004016
SBP, BMI AND PP	2	rs848286	58007905	58674393
CAD, BMI AND PP	2	rs72932707	203639395	204196618
HDL AND DBP	2	rs6738482	61242410	61837947
WHR AND DBP	2	rs17741344	148457576	148853296
TC AND LDL	3	rs9853387	135798730	136503896
CAD, BMI, HDL, LDL, TC AND PP	3	rs6788993	52277445	52838402
SBP, BMI AND PP	3	rs12637791	85403892	85784084
TC, DBP AND SBP	3	rs1989839	50184538	50420554
CAD, DBP AND SBP	4	rs4691707	156420605	156443314
SBP AND PP	4	rs16854051	41879969	42161491
BMI, DBP AND SBP	4	rs11722027	144028173	144215346
CAD, T2D AND DBP	4	rs17516389	118976252	119264162
BMI AND PP	5	rs4269288	122650049	122803786
T2D, DBP, SBP AND PP	6	rs10948071	43260660	43397259
WHR, TG, LDL, CRP AND PP	6	rs2856674	25450026	32963948
HDL, CRP, LDL, TC, TG AND SBP	6	rs2269426	31578772	32189481
CAD, WHR, CRP AND SBP	6	rs1490384	126659043	127080700
CAD, HDL, LDL, WHR, BMI, CRP AND DBP	6	rs1077393	30997692	32189481
T2D AND DBP	6	rs2396004	43262303	43364494
CAD, WHR, CRP, T2D AND DBP	6	rs6925689	126623947	127080700
TC AND LDL	7	rs6948810	21474610	21555536
SBP AND PP	7	rs17165701	12212919	12286050
CAD AND SBP	7	rs58673065	1843200	2110850
CAD AND SBP	7	rs6460902	12200060	12285140
DBP, HDL, BMI, LDL AND PP	8	rs7813434	116464988	116632819
CRP, DBP, SBP AND PP	8	rs2736313	8088230	12199830
WHR AND PP	9	rs11791636	23805555	23827667
SBP AND PP	9	rs10821154	96155812	96381916
SBP AND PP	9	rs4838254	127766897	128399285
SBP, CAD, BMI AND PP	10	rs11000925	75867193	76421529
T2D, DBP AND SBP	10	rs10906382	13479684	13611368
CAD, BMI, DBP, SBP AND PP	10	rs77335224	104487871	105059896
BMI, LDL AND TC	11	rs866901	77909014	78135704
HDL OG TG	11	rs10832027	13354509	13370535
SBP, BMI AND PP	11	rs3180446	45203212	45345244
SBP, CAD, BMI, LDL,TC AND PP	11	rs2450122	77909014	78135704
HDL AND SBP	11	rs1988724	9958403	10370675
BMI AND SBP	11	rs11038371	45258966	45345244
BMI, T2D, CRP, TC, TG, HDL, LDL, PP, DBP, AND SBP	11	rs7107356	47175327	49128599
CAD, BMI, LDL, TC AND SBP	11	rs990706	77909014	78271614
SBP AND PP	12	rs79601649	49737114	50160662
HDL OG TC	14	rs12588415	75120628	75378185
TC, SBP AND PP	14	rs1866628	75057809	75113506
HDL,TC AND DBP	14	rs8004084	75144618	75377692
BMI AND PP	15	rs4886937	78076272	78152626
CAD, SBP, DBP AND PP	15	rs17514846	91412850	91429042
BMI AND SBP	15	rs7176782	69415482	69569464
CAD AND SBP	15	rs17514846	91412850	91429042
TC AND LDL	16	rs1002252	71278016	71376751
BMI, TG AND DBP	16	rs1549299	30916129	31155458
TC AND LDL	17	rs12309	38122708	38219005
TC AND LDL	17	rs1230065	43461460	43534322
SBP AND PP	17	rs2165846	44941366	44947821
CAD AND DBP	17	rs55938136	43798360	43798360
CAD, BMI, WHR, HDL	18	rs17700144	57728947	57987859
HDL OG TG	19	rs10409835	32830261	32994338
BMI AND WHR	19	rs9636202	18449238	18474892

Z in NEUR	Nearest gene
4.35461662317	RPL31P12
4.18963785615	KCNK3
−3.89234482431	CAMKMT
5.45230886725	FANCL
4.13516480072	ICA1L
4.45360597614	USP34
5.54325499552	ACVR2A
−4.98578731358	PCCB
4.29741691262	SMIM4:PBRM1
−5.00576709809	CADM2
−5.10311943356	ZMYND10-AS1:ZMYND10
−4.35330561706	MTND1P22
−5.43567296604	BEND4
3.94478982621	RP11–284M14.1
−4.33016153627	PRSS12
−4.89325849576	CEP120
−4.08127148845	ZNF318
5.47679437498	MTCO3P1
7.85464688518	TNXB:ATF6B
−5.2537909447	MIR588
−5.66008277462	BAG6
3.72892286408	ZNF318
4.68322226982	RNU6–200P
5.17023483889	SP4
−6.05105534044	TMEM106B
4.4437848434	MAD1L1
5.92306285077	TMEM106B
4.17514218028	TRPS1
−5.84975419193	LINC00529
−4.68062619372	ELAVL2
5.23143409848	FAM120A
−4.98786564489	HSPA5
−4.11173316994	ADK
−4.36400954759	BEND7
−5.23859919803	C10orf32-ASMT:AS3MT
−4.31225213337	GAB2
−5.43398246143	ARNTL
−4.74001727338	SYT13
4.27353276097	GAB2
4.6699316277	SBF2
4.02407371564	SYT13
−7.04747006968	AGBL2
−4.17713795565	RP11–452H21.4
4.76020126525	SPATS2
6.86308309352	YLPM1
4.64137402432	LTBP2
−6.54353852104	YLPM1
5.66999702405	LINGO1
−4.43830518151	FURIN
−3.94284707938	GLCE
−4.43830518151	FURIN
−5.2061488614	HYDIN
4.47612498022	PRSS36
−4.91571280463	MED24
−6.04762128386	ARHGAP27
4.77322394436	WNT9B
−11.5419461181	CRHR1:RP11–105N13.4
−5.50787070534	RPS3AP49
−5.29319139928	AC007773.2
−3.89258923647	PGPEP1

*BP* base position, *CHR* chromosome, *NEUR* neuroticism, *CAD* coronary artery disease, *BMI* body mass index, *WHR* waist-hip-ration, *HDL* high density lipoprotein (HDL), *LDL* low density lipoprotein, *TG* triglycerides, *TC* total cholesterol, T2D, *CRP* c-reactive protein, *SBP* systolic blood pressure, *DBP* diastolic blood pressure, *PP* pulse-pressure, *CIGPRDAY* cigarettes smoked per day.

### Effect directions

Of the top lead SNPs (conjFDR < 0.05) shared between neuroticism and CAD, 21 (70%) had the same direction of effect, 18 (81.8%) for WHR, 36 (60%) for SBP, and 28 (60%) for DBP, which implies that the genetic variants increase risk for both neuroticism and CAD, WHR, SBP, and DBP, respectively. For neuroticism and HDL, 16 (55%) of the identified loci had opposite effect directions, as could be expected because higher HDL is associated with lower risk for CAD^51^. However, for neuroticism and BMI, 56 (58%) of the top lead SNPs also showed the opposite effect direction, suggesting mixed effect directions, with a tendency for neuroticism risk to be somewhat associated with reduced BMI. For the other CVD risk factors, there was a mixed patterns of effect directions. The effect directions are similar to the polygenic effect directions from the genetic correlation analyses (Suppl. Fig. 1).

### Functional analyses

Functional annotations of all SNPs having a conjFDR < 0.05 for neuroticism versus CAD and CVD risk factors are shown in Supplementary Tables 1–13. The shared loci implicated genes associated with pathways of cell division and proteasome degradation for CAD, starch, and sucrose metabolism for BMI and HDL, and nuclear receptor transcription for HDL, among others. Finding of involvement of the nuclear receptor transcription pathway is in line with recent evidence, that activation of the nuclear receptor FXR in vivo increases hepatic levels of miR-144 and lowers hepatic ABCA1 and plasma HDL levels^52^. For SBP and PP the shared loci implicated genes associated with elastic fiber formation pathways, and for DBP the shared loci implicated genes associated with the Notch signaling pathway, among others.

## Discussion

The present results demonstrated extensive overlapping polygenic architecture between neuroticism and CVD risk factors and CAD beyond genetic correlation. We identified 345 unique genetic loci underlying the shared genetic architecture, and increased the number of loci associated with neuroticism to *n* = 729, due to the boost in power from combined analysis of GWAS from two phenotypes using the cond/conjFDR method. This provides new knowledge about the molecular genetic mechanisms shared between cardiovascular risk and neuroticism.

We identified 345 genetic variants jointly associated with neuroticism and CVD risk factors as well as CAD; 30 for CAD, 96 for BMI, 46 for DBP, 60 for SBP, 22 for WHR, and 29 for HDL, as well as between 9–36 for each of PP, T2D, TG, TC, LDL, CRP, and one for CIGPRDAY. These low number of shared loci between neuroticism and smoking compared to BMI and blood pressure is probably due to the lower polygenicity of smoking. Although the initial GWASs had reasonably same statistical power, the number of significant loci were much lower in the original smoking GWAS (*n* = 3)^27^, compared to the original BMI GWAS (*n* = 423)^22^, and blood pressure GWAS (*n* = 505)^26^.

While some tag SNPs may represent the same causal locus, 10, 17, 19, 9, 15, and 29 were novel for CAD, BMI, DBP, SBP, WHR, HDL, respectively. The effect direction was mostly positively concordant for neuroticism and CAD, WHR, SBP, and DBP, whereas it was mostly negatively concordant for neuroticism and BMI and HDL. This is in line with PRS and genetic correlation between neuroticism and CAD and CVD risk factors in earlier studies^1,18^ However, the genetic correlations are weak, and significant only for CAD, WHR, and TG. This suggests that there is an overall increased genetic risk for CAD associated with neuroticism at the group level. Yet, the conjFDR analysis reveals multiple shared loci with both same and opposite effect directions, indicating a more complex genetic relationship underlying these phenotypes than what is captured by the genetic correlations; some individuals may have genetic variants that increase risk to both neuroticism and CVD, while others have the opposite direction, and some a mix of both directions^53^. Thus, this seems to indicate the presence of subgroups of neuroticism with specific increased vulnerability to certain CVD risk factors.

Interestingly, there was an negative genetic association between BMI and neuroticism, which implicates that most gene loci associated with lower BMI are associated with higher scores on neuroticism. This seems to be opposite of findings with regards to neuroticism and CAD and WHR. A possible explanation is that WHR is a better marker of central obesity, total fat, or fat distribution than BMI^54^ and thus better correlated with CAD outcome. There is also some evidence indicating that activation of the sympathetic nervous system and release of neuroendocrine hormones, cytokines and inflammatory markers from adipocytes among patients with central obesity may be linked to neuroticism^55^. In our study, we also found some loci shared between neuroticism and other CVD risk factors, including lipids (HDL, LDL, TC, and TG), blood pressure (PP), T2D and CRP, also here suggesting a mixed genetic pattern of effects. As far as we are aware, only one study has tested for shared genes between HDL, LDL, and neuroticism and they did not find significant associations^18^. No significant associations have previously been found between PGR for SBP, DBP, and T2D and neuroticism^1^. In the same study, higher PGR for smoking was associated with higher levels of neuroticism^1^. However, we did not find an association between neuroticism and CIGPRDAY in the present study. To the best of our knowledge, we are the first to investigate genetic overlap between TC, TG, CRP, and neuroticism.

The large shared polygenic signal between neuroticism and CAD, BMI, WHR, and HDL may suggest underlying metabolic mechanisms for both CAD development and neuroticism. The involvement of the starch and sucrose metabolism pathway in BMI and HDL may support this. Yet, only 70% of the associated genetic variants showed concordant effects on neuroticism and CAD risk, suggesting a more complex genetic interplay. For HDL, our analyses also revealed loci mapped to genes encoding for nuclear receptor transcription. Finding of involvement of the nuclear receptor transcription pathway is in line with recent evidence, that activation of the nuclear receptor FXR in vivo increases hepatic levels of miR-144 lower hepatic ABCA1 and plasma HDL levels^52^. For CAD, gene set analyses revealed involvement of the cell division pathway. Recent advances in research to prevent restenosis in CAD patients focus on antiproliferative strategies that target the cell cycle^51^. Further, gene set analyses implicated involvement in the proteasome degradation pathway for CAD. Exciting progress in elucidating the pathophysiological significance of protein degradation and protein quality control in heart diseases has occurred in the past several years^56^. Alterations in cardiac proteasomal degradation are linked with most heart diseases, including CAD^57^. Rapidly mounting evidence suggests that the proteasome may be a therapeutic target for heart disease^58^. For SBP and PP the shared loci with neuroticism implicated genes associated with pathways of elastic fiber formation. Elastic fibers might be key elements in the pathophysiology of hypertensive vascular remodeling. They are composed of elastin and multiple other heterogeneous components and they are mainly responsible for extensibility and resilience of tissues. In the circulatory system, the proper assembly and functioning of elastic fibers is absolutely crucial for maintaining a smooth and uninterrupted delivery of blood from the heart to organs and tissues^59^. It is well-established that structural and mechanical abnormalities leading to large artery stiffening and resistance artery narrowing are two of the main features associated with essential hypertension, which, in the end, is deleterious for cardiovascular function^60^. The question has been whether structural alterations in the arterial wall in hypertension are a consequence of disease or early cellular alterations, determined genetically or by environmental factors^59^. Here we provide evidence suggesting the involvement of genetic factors. In line with this, genetic defects of elastic fiber components have previously been associated with abnormal vessel structure and hypertension^61,62^.

The shared loci between DBP and neuroticism implicated genes involved in the Notch signaling pathway. Recently, the hypothesis that Notch signaling controls the expression of soluble guanylyl cyclase, the major nitric oxide receptor in the vascular wall in vascular smooth muscle, was addressed. Reduction of nitric oxide -dependent vasodilatation in hypertension is due in part to a reduction of the protein level of soluble guanylyl cyclase^63^. However, the above discussed possible common pathophysiological mechanisms for neuroticism and CAD are somewhat speculative, and experimental studies are needed to better understand mechanisms related to the shared genetic loci identified in the current study.

In the original neuroticism GWAS a total of 136 genome-wide loci were reported^14^. By conditioning the original neuroticism GWAS (*n* = 432,109 participants) on the CAD and CVD risk factors GWAS (*n* = 184,305–339,224 participants), we identified 729 unique loci associated with neuroticism. Thus, over 500 of these loci were not reported in the original neuroticism GWAS. This provides new information about the molecular factors underlying this core human mental trait, which is associated with several psychiatric diagnostic categories^2,64^. Further, these findings illustrate how the combined analyses of two GWAS can boost the power to identify loci if there is shared polygenic architecture^19^. The current findings further establish neuroticism as a polygenic trait, with potential for revealing more of the underlying genetic loci if larger samples are investigated^65^.

Despite the finding that high neuroticism predicts poor outcome on CAD^5,6^, it is not established practice to screen for neuroticism in patients with CAD or CVD risk factors. When genetic tests become more affordable, testing for genetic CAD risk may be cost effective, and implemented as a part of risk assessment in routine clinical practice. This will give patients the possibility to reduce their risk profile through lifestyle changes such as diet and exercise, and allow for closer follow-up from their physician many years in advance of developing CAD, which may have great impact on prognosis.

Strengths of the present study include that we combined samples from UK Biobank and 23andMe to obtain a large sample size, and that we used an established method, which provides increased power to detect novel genetic loci^19^. There are certain limitations to the present results; as our analyses were restricted to people with European ancestry the results need to be replicated in those with different genetic background to be generalized to different populations. Further, many variables are self-reported and measured at only one occasion. Also, due to the inability to identify the causal variants from GWAS, we cannot rule out that different tag SNPs can represent the same causal locus.

In conclusion, the present study shows substantial polygenic overlap between neuroticism, CAD and CVD risk factors, most strongly with BMI, DBP, SBP, WHR, and HDL, and identified 345 genetic loci underlying the shared genetic architecture.

## Supplementary information

Supplementary Table 1-15

Supplementary Figure 1-32

## Data Availability

This study used openly available software and code, specifically LD-score regression [https://github.com/bulik/ldsc/] and conjunctional FDR [https://github.com/precimed/pleiofdr/].
