# Supplementary material for: Shared genetic architecture between neuroticism, coronary artery disease and cardiovascular risk factors
Source: Transl Psychiatry. 2021 Jun 17;11:368. doi: 10.1038/s41398-021-01466-9 (PMC8257646; doi:10.1038/s41398-021-01466-9)
Supplement: Supplementary file 1 — Supplementary Table 1-15 [file 41398_2021_1466_MOESM1_ESM.pdf]

Phenotype	Number of individuals (n)	Age (years)	Ancestry	Recruitment	Ref.
**Neuroticism**	432128	16-85	European	UKBB=372903 23andME=59225	(1)
**CAD**	184305	>18	77% of European ancestry; 13% and 6% of south (India and Pakistan) and east (China and Korea) Asian ancestry with smaller samples of Hispanic and African Americans	Cases=60801 Controls=123504 From 48 studies.	(2)
**BMI**	339224	Mean (SD) 19.1 (0.57) - 75.7 (3.40)	European	From 125 studies, 82 with GWAS results (*n* = 236231) and 43 with results from Metabochip (*n* = 103047)	(3)
**TC**<br>**TG**<br>**LDL**<br>**HDL**	188577	Mean (SD) 16 (0.4) - 75 (10.2)	European	94595 individuals from 23 studies genotyped with GWAS arrays and 93982 individuals from 37 studies genotyped with the Metabochip array	(4)
**SBP**<br>**DBP**	69395	Mean (SD) 19.1 (3.6) - 72 (5)	European	From 29 studies	(5)
**T2D**	159208	Mean (SD) 39.71 (16.07) - 73.5 (12)	European	Cases=26676 Controls=132532. Findings were followed up in additional independent	(6)

				European ancestry studies of 14545 T2D case and 38994 control subjects genotyped using the Metabochip<br><br>From 18 studies genotyped using commercial genome-wide single nucleotide variant (SNV) arrays. The Metabochip stage 2 follow-up comprises 14545 T2D case and 38994 control subjects (N_{eff} = 38,645) from 16 nonoverlapping stage 1 studies. Additional follow-up in 2796 T2D case and 4601 control subjects from the European Prospective Investigation into Cancer and Nutrition-InterAct (EPIC-InterAct) study and in 9747 T2D case and 61857 control subjects from the Resource for Genetic Epidemiology on Adult Health and Aging (GERA) study.	
**WHR**	224459	17-97	European	142762 individuals with GWAS data and 81697 individuals	(7)

				genotyped with the Metabochip, all from the Genetic Investigation of ANthropometric Traits (GIANT) Consortium.	
**CRP**	204402	18- >65	European	From 88 studies	(8)
**CIGPRDAY**	74053	21-102	European	From 16 studies	(9)

Suppl. Table 14

1. Nagel M, Jansen PR, Stringer S, Watanabe K, de Leeuw CA, Bryois J, et al. Meta-analysis of genome-wide association studies for neuroticism in 449,484 individuals identifies novel genetic loci and pathways. Nat Genet. 2018;50(7):920-7.
2. Nikpay M, Goel A, Won HH, Hall LM, Willenborg C, Kanoni S, et al. A comprehensive 1,000 Genomes-based genome-wide association meta-analysis of coronary artery disease. Nat Genet. 2015;47(10):1121-30.
3. Wood AR, Esko T, Yang J, Vedantam S, Pers TH, Gustafsson S, et al. Defining the role of common variation in the genomic and biological architecture of adult human height. Nature Genetics. 2014;46:1173.
4. Global Lipids Genetics C, Willer CJ, Schmidt EM, Sengupta S, Peloso GM, Gustafsson S, et al. Discovery and refinement of loci associated with lipid levels. Nature Genetics. 2013;45:1274.
5. Ehret GB, Munroe PB, Rice KM, Bochud M, Johnson AD, Chasman DI, et al. Genetic variants in novel pathways influence blood pressure and cardiovascular disease risk. Nature. 2011;478(7367):103-9.
6. Scott RA, Scott LJ, Mägi R, Marullo L, Gaulton KJ, Kaakinen M, et al. An Expanded Genome-Wide Association Study of Type 2 Diabetes in Europeans. Diabetes. 2017;66(11):2888-902.
7. Shungin D, Winkler TW, Croteau-Chonka DC, Ferreira T, Locke AE, Magi R, et al. New genetic loci link adipose and insulin biology to body fat distribution. Nature. 2015;518(7538):187-96.
8. Ligthart S, Vaez A, Võsa U, Stathopoulou MG, de Vries PS, Prins BP, et al. Genome Analyses of >200,000 Individuals Identify 58 Loci for Chronic Inflammation and Highlight Pathways that Link Inflammation and Complex Disorders. The American Journal of Human Genetics. 2018;103(5):691-706.
9. Furberg H, Kim Y, Dackor J, Boerwinkle E, Franceschini N, Ardissino D, et al. Genome-wide meta-analyses identify multiple loci associated with smoking behavior. Nature Genetics. 2010;42(5):441-7.

locusnum	CHR	LEAD_SNP	LEAD_BP	MinBP	MaxBP
1	1	rs4845681	154868055	154834092	154980351
2	1	rs71631900	197422656	197422656	197422656
3	1	rs10800792	201811144	201797696	201887382
4	2	rs11675585	24237180	23933132	24627074
5	2	rs72932707	203639395	203563940	204385122
6	2	rs16852064	215447252	215439708	215515697
7	3	rs3819325	49843723	49734229	49960388
8	3	rs1529544	53039455	52962681	53171555
9	3	rs3772912	81698481	81630790	82022078
10	3	rs9877786	136385519	135625498	136638975
11	4	rs17626479	119129455	118976252	119264162
12	4	rs4691707	156441314	156420605	156443314
13	5	rs185935	107457354	107346393	107769562
14	6	rs2395476	31327382	31252396	31346898
15	6	rs1591805	126717064	126623947	127080700
16	7	rs58673065	1885600	1858467	2090849
17	7	rs11509880	12261911	12173173	12285329
18	10	rs73284278	76011751	75867193	76421529
19	10	rs77335224	104636276	104487871	105059896
20	11	rs34862781	13305263	13268386	13370535
21	11	rs2512527	77920863	77919768	77968631
22	14	rs1204996	72186684	72168695	72218345
23	14	rs56287046	75621020	75519193	75628199
24	15	rs17514846	91416550	91412850	91429042
25	16	rs13332167	30557226	30557226	30557226
26	17	rs2350633	17587395	17519037	17644405
27	17	rs8072451	43893716	43463493	44865603
28	18	rs17700144	57811982	57728947	57913434
29	20	rs2295096	33464710	33288002	33880146
30	22	rs13053618	31457195	31440703	31491163

Overlapping BP with	A1	A2	FDR	Z_in_NEUR	Z_in_CAD
	T	G	0.020189244	4.12205960835	3.952311174
BMI	A	G	0.042387577	4.33343595574	-3.40320618
BMI,CIGPRDAY	A	G	0.044433537	-4.01737513633	3.386578602
WHR	T	C	0.021138216	4.99137814161	-3.62938251
PP	T	C	0.019441868	4.13516480072	-8.34419598
	T	C	0.005543128	-5.47992756462	-3.99792570
	T	C	0.043369644	-3.86940786421	-3.39523387
BMI,LDL,PP	A	G	0.035813590	4.57031141156	-3.46053657
	A	G	0.041836774	4.33904994681	3.407670336
TC,TG	A	G	0.001230664	-5.07856316372	-4.34229816
DBP	A	C	0.010993223	4.32360504146	4.056094860
SBP	A	G	0.009981500	-4.35330561706	-4.99283300
BMI, HDL	A	G	0.044703784	5.55747829897	-3.38437690
BMI,TC,DBP	T	G	0.049815955	3.79389902841	3.362250340
WHR,CRP,DBP	A	G	0.003532297	4.71840578957	-4.10632716
SBP	A	G	0.049820827	4.4437848434	3.346529405
SBP,PP	A	G	0.004619870	6.25097430058	4.042873989
SBP	T	C	0.045566248	3.84006847133	-3.37734195
BMI, DBP,SBP,PP	T	C	0.000549599	-5.23859919803	5.668563145
TG	A	G	0.003239406	-6.7288578552	-4.12732972
LDL,BMI,SBP,PP	A	C	0.027745550	-4.01175120052	3.644799828
	A	C	0.030281970	-4.93195661768	-3.51594000
	A	G	0.031951013	-4.13829257749	3.498034783
SBP,DBP,PP	A	C	0.007687090	-4.43830518151	-5.11726374
	T	C	0.047412433	-3.86213591789	-3.36357473
	A	G	0.009310208	4.37704344936	3.935568457
DBP,LDL	T	C	0.034891503	11.5524327173	-3.46910259
TG,CRP,HDL,T2D	A	G	0.000192184	-5.50787070534	-4.82077719
TG	A	G	0.040110248	-3.87712053537	3.579539667
	A	G	0.014638721	4.22874357809	3.824195090

nearestGene	dist	func	CADD	RDB	minChrState	PVAL_in_NE
KCNN3	25298	intergenic	3.578	5	5	3.755e-05
CRB1	0	intronic	0.112	5	5	1.468e-05
IPO9	0	intronic	0.877	6	4	5.885e-05
MFSD2B	0	intronic	1.761	6	4	5.995e-07
ICA1L	1294	upstream	4.065	5	4	3.547e-05
VWC2L	3568	ncRNA_intro	1.176	5	5	4.255e-08
UBA7	0	intronic	1.486	NA	2	0.0001091
SFMBT1	0	intronic	1.972	NA	5	4.87e-06
GBE1	0	intronic	5.307	NA	4	1.431e-05
STAG1	0	intronic	0.504	6	5	3.803e-07
NDST3	0	intronic	12.73	5	4	1.535e-05
MTND1P22	56338	intergenic	0.347	7	9	1.341e-05
FBXL17	0	intronic	1.509	NA	5	2.737e-08
HLA-B	2416	intergenic	5.617	5	2	0.0001483
CENPW	47042	intergenic	4.04	NA	5	2.377e-06
MAD1L1	0	intronic	4.431	5	2	8.839e-06
TMEM106B	0	intronic	2.275	6	4	4.079e-10
ADK	0	intronic	11.25	6	5	0.000123
C10orf32-AS	00:00	intronic	3.704	7	4	1.618e-07
ARNTL	0	intronic	1.501	4	2	1.71e-11
USP35	0	exonic	0.05	NA	4	6.027e-05
SIPA1L1	0	intronic	0.881	NA	4	8.141e-07
TMED10	0	intronic	0.495	7	5	3.499e-05
FURIN	0	intronic	10.53	4	1	9.067e-06
ZNF764	7858	intronic	4.463	7	5	0.0001124
RAI1	0	intronic	8.545	4	2	1.203e-05
CRHR1:RP11-	00:00	ncRNA_intro	0.262	1f	5	7.176e-31
RPS3AP49	4825	ncRNA_intro	3.068	6	7	3.632e-08
ACSS2	0	intronic	11.23	2b	1	0.0001057
RN7SL633P	925	downstream	0.72	4	5	2.35e-05

BETA_in_NE	SE_in_NEUR	PVAL_in_CA	BETA_in_CA	SE_in_CAD
0.0096	0.0023	7.74e-05	0.037621	0.0095191
0.2753	0.0635	0.000666	-0.046303	0.0136056
-0.0091	0.0023	0.0007077	0.031608	0.0093333
0.0117	0.0023	0.0002841	-0.034986	0.0096396
0.014	0.0034	7.17e-17	-0.130189	0.0156022
-0.0181	0.0033	6.39e-05	-0.052753	0.013195
-0.0088	0.0023	0.0006857	-0.032242	0.0094962
0.0105	0.0023	0.0005391	-0.032024	0.009254
0.0102	0.0024	0.0006552	0.032945	0.0096679
-0.0121	0.0024	1.41e-05	-0.044907	0.0103435
0.0167	0.0039	4.99e-05	0.065442	0.0161345
-0.0104	0.0024	5.95e-07	-0.04757	0.0095277
0.0135	0.0024	0.0007134	-0.037036	0.0109432
0.009	0.0024	0.0007731	0.039723	0.0118144
0.0107	0.0023	4.02e-05	-0.040166	0.0097808
0.012	0.0027	0.0008183	0.03548	0.0106021
0.0151	0.0024	5.28e-05	0.039003	0.0096472
0.013	0.0034	0.0007319	-0.046971	0.0139076
-0.0212	0.004	1.44e-08	0.08313	0.0146658
-0.0154	0.0023	3.67e-05	-0.039457	0.0095592
-0.0134	0.0033	0.0002676	0.04742	0.0130104
-0.0112	0.0023	0.0004382	-0.035542	0.0101089
-0.0104	0.0025	0.0004687	0.038918	0.0111257
-0.0102	0.0023	3.1e-07	-0.050925	0.0099514
-0.012	0.0031	0.0007694	-0.045844	0.0136295
0.0099	0.0023	8.3e-05	0.039971	0.0101567
0.0314	0.0027	0.0005222	-0.046755	0.0134775
-0.0153	0.0028	1.43e-06	-0.05689	0.0118024
-0.0103	0.0027	0.0003442	0.038133	0.0106531
0.0146	0.0035	0.0001312	0.054893	0.0143541

locusnum	CHR	LEAD_SNP	LEAD_BP	MinBP	MaxBP	Overlapping
1	1	rs7535528	2444414	2408661	2444414	
2	1	rs11120813	7062993	7061430	7128056	
3	1	rs11121210	8708529	8457648	8895970	PP
4	1	rs11211481	47694167	47659445	47708112	
5	1	rs6667780	49335330	49228284	49402084	
6	1	rs1460940	72814617	72628347	72959392	TG,WHR
7	1	rs1339429	97141811	97115049	97283948	
8	1	rs6428020	190282725	190132619	190310334	
9	1	rs2488401	197702401	197342380	197813558	CAD
10	1	rs1022361	201790270	201760610	201919960	CAD,CIGPRD
11	1	rs7549696	210077605	210068117	210307185	
12	2	rs1275988	26914364	26911509	26932796	SBP,DBP,PP
13	2	rs11682175	57987593	57917222	58505679	SBP,PP
14	2	rs10175173	64815339	64732145	64820430	
15	2	rs2436075	157136419	157014004	157150188	
16	2	rs12373663	164591887	164506159	164600455	
17	2	rs6738445	172599615	172521827	172911094	
18	2	rs1541853	203835177	203639395	204196618	PP
19	2	rs11894483	237114619	237105050	237143805	
20	3	rs11128818	17143621	16973762	17151126	
21	3	rs9820537	25092101	25077259	25144648	
22	3	rs10514710	44883971	44762830	44883971	
23	3	rs2014830	50172397	49980596	50172397	
24	3	rs6794389	53096394	52277445	53101580	CAD,LDL,T2D
25	3	rs863635	61262414	61159033	61277928	
26	3	rs4353776	70528794	70456975	70536179	
27	3	rs3772882	81808602	81630790	82022078	CAD,HDL
28	3	rs7611991	85759558	84964976	86207903	SBP,PP
29	3	rs6804355	116981483	116956205	117009819	
30	3	rs6786582	135898984	135625498	136638975	CAD,TG,TC,L
31	3	rs827172	157958717	157829953	158284861	
32	4	rs6822869	143891507	143786837	144215346	SBP,DBP
33	5	rs12514697	64855976	64779214	65006234	
34	5	rs1501672	87963761	87936379	87988934	WHR
35	5	rs10036343	107626554	107389947	107714202	CAD,HDL
36	5	rs13159814	115602323	115422545	115646929	
37	5	rs11241696	122759862	122650049	122766938	PP
38	5	rs17536714	153526051	153497584	153573938	
39	5	rs4481363	164474719	164465319	164763768	
40	6	rs9380238	31267618	31236201	31319606	CAD,TC,LDL,
41	6	rs6925748	50930041	50930041	50930041	
42	6	rs9401491	98481904	98310091	98748005	
43	6	rs10457800	100936628	100890654	101044487	TC

44	6	rs9492443	130398731	130374461	130422142	TG
45	6	rs7739842	131954797	131897278	131973932	
46	7	rs7811417	21534152	21470536	21552995	TC,LDL
47	7	rs12704648	92652339	92646881	92671276	
48	7	rs11768389	127150250	127059236	127214967	
49	7	rs1593304	131619847	131619847	131627573	
50	8	rs2409722	11039816	10312602	11450422	TG
51	8	rs13263851	14164646	14041437	14164856	
52	8	rs1366968	107083663	106970575	107094661	
53	8	rs2293889	116599199	116464988	116639474	HDL,LDL,DBP
54	9	rs7045135	11421771	11274206	11887262	
55	9	rs997318	37364094	37073902	37379492	
56	9	rs16909859	98204792	98153936	98278413	
57	9	rs10491851	120444525	120444525	120474282	
58	9	rs7045289	129913558	129625226	129948771	
59	10	rs7097384	9523803	9436362	9585398	
60	10	rs2279648	75867193	75867193	76421529	PP
61	10	rs7098825	104628234	104487871	105059896	CAD,SBP,PP
62	10	rs993743	107517078	107491387	107564172	
63	11	rs9633835	13345593	13268386	13350131	CAD,TG
64	11	rs3026401	31807524	31807524	31813529	
65	11	rs2863181	45295347	45295347	45448520	SBP,PP
66	11	rs7103648	47461783	47229316	48489360	TC,HDL,LDL,T[illegible]
67	11	rs866901	77926309	77909014	78135704	CAD,LDL,TC,[illegible]
68	11	rs1452134	86133416	86111463	86166928	
69	11	rs10891496	112920921	112826709	113049801	
70	11	rs2512885	131467794	131463916	131483597	
71	12	rs7962050	1031454	875653	1051867	
72	12	rs651548	99560183	99436519	99639930	
73	12	rs4766615	110060130	110060130	110060130	LDL
74	13	rs1045411	31033232	31012904	31043841	
75	13	rs3105038	55968795	55679499	56303709	
76	13	rs7989314	59283765	59104084	59301167	
77	13	rs4884332	59900365	59899311	59927107	
78	13	rs4483743	68195416	68142842	68195416	
79	13	rs2590556	94110198	94020716	94116448	
80	15	rs9944219	46500612	46305384	46586095	
81	15	rs745213	68060389	67680209	68140315	WHR
82	15	rs7176782	69477380	69415482	69649385	SBP
83	15	rs7164558	78146382	78044631	78159256	PP
84	16	rs7294	31102321	30897456	31155458	TG,DBP
85	17	rs4075482	79074548	79046374	79093822	
86	18	rs11081818	31251088	31186985	31298923	
87	18	rs7227409	35336058	35288227	35414690	

88	18	rs9953197	50047651	50037650	50148717	
89	18	rs12953492	52527465	52471537	52569120	
90	18	rs17700144	57811982	57728947	57987859	CAD,TG,CRP,
91	19	rs9636202	18449238	18449238	18474892	WHR
92	19	rs1469712	19528821	19358672	19657632	
93	19	rs8101491	47642780	47637608	47645777	
94	20	rs6058089	33194257	32933747	33525407	CAD,TG
95	21	rs2836753	40291187	40289167	40316902	
96	22	rs926914	41418154	41085969	41713111	CRP

A1	A2	FDR	Z_in_NEUR	Z_in_BMI	nearestGene	dist
A	G	0.016459835	4.204292564	-4.15910286	PANK4	0
A	G	0.041479069	-4.18306904	2.924960453	CAMTA1	0
T	C	0.014383712	4.260796112	-3.52630585	RERE	0
A	G	0.047929325	3.705949815	4.315835995	TAL1	0
T	C	0.031260115	3.918342386	3.219532064	AGBL4	0
A	G	0.011390901	4.354616623	-9.37536015	RPL31P12	47104
T	C	0.039774068	3.801711494	-3.62158815	RPL7P9	2618
A	G	0.012874904	4.306310509	3.94593252C	BRINP3	0
T	C	0.014739649	-6.38164126	3.326544432	DENND1B	0
A	G	0.001396517	5.090139601	-4.09757550	NAV1:IPO9-A	00:00
A	G	0.024953845	4.024197194	-3.23252825	SYT14	33932
T	C	0.017672565	-4.17552469	4.131566132	KCNK3	1254
T	C	0.003731351	-5.23220482	-3.73690401	VRK2	147192
A	G	0.040625086	4.093887605	2.934393704	AFTPH	0
A	G	0.018387019	-6.45928331	3.250012342	NR4A2	44524
T	C	0.034905725	-4.12827175	-2.99997699	FIGN	0
T	C	0.021426327	4.092308456	3.39435682C	DYNC1I2	0
A	C	0.039458768	3.839271329	-2.99716855	WDR12:CARF	00:00
T	C	0.031889979	-3.91103881	3.092617012	ASB18	0
T	C	0.020848639	-4.34339116	-3.20406899	PLCL2	11534
T	C	0.020940686	-4.10239241	-3.78566188	RNA5SP125	25483
A	G	0.026359049	-4.23544453	3.114591204	KIF15	0
T	C	0.040602119	-3.79123505	3.972876066	RP11-493K19	0
A	G	0.009573005	4.547643225	-3.46659267	SERBP1P3	1904
A	G	0.037150999	-3.83473449	4.783813833	FHIT	25280
T	C	0.046749111	3.768699957	-2.95124086	RP11-231I13	166916
A	C	0.006171574	-4.58823933	4.052653392	GBE1	0
A	G	0.002521938	-4.90133000	-6.35702389	CADM2	0
T	C	0.031116783	-4.07285669	-3.04756972	LSAMP	0
T	C	0.004218642	-4.72483700	-4.66434605	MSL2	0
A	G	0.038488553	-3.84146936	-3.02699260	RSRC1	0
A	G	0.030842822	-3.92511031	3.162724816	RP11-284M1	0
T	G	0.043372704	3.88626113C	-2.90478964	CENPK	0
A	G	0.001722977	-6.23316645	3.935539526	LINC00461	0
A	C	0.042020956	5.457789025	-2.91896114	FBXL17	0
A	C	0.026942039	-4.22288023	3.105589721	COMMD10	0
T	C	0.034905725	-4.11285255	-2.99997699	CEP120	575
A	G	0.012877068	4.626498254	-3.37209024	MFAP3	0
A	C	0.005146776	4.89173689C	3.648671409	CTC-340A15.	0
A	G	0.01018453C	-4.45523845	3.446451742	WASF5P	10876
A	G	0.004883281	-4.67246930	-3.73690401	FTH1P5	49071
A	G	0.034905725	6.087761871	2.999976992	RP11-436D23	0
T	C	0.032942405	4.834918793	3.024068454	ASCC3	19441

T	C	0.022080475	-4.07869092	-3.27974758	L3MBTL3	0
T	G	0.01374659	4.27892277	-3.60413022	ENPP3	0
T	C	0.033031521	4.838382567	-3.02297853	SP4	0
A	G	0.03978772	3.87804325	-2.94361812	RN7SL7P	51725
T	C	0.012363202	-4.32231576	-3.67259483	AC000124.1	24391
A	G	0.01237563	-5.03477917	3.38460788	AC009518.4	0
T	G	0.032643012	-4.19134958	-3.02772770	XKR6	0
A	C	0.006107195	4.73878827	-3.60001403	SGCZ	0
T	C	0.04733056	3.784690474	2.88938187	SLC16A14P1	144327
T	G	0.01532982	-4.80584403	3.31360961	TRPS1	0
T	C	0.014072134	5.716250763	3.34209031	IMP3P1	591462
T	C	0.04493051	-3.73961816	-3.63157203	ZCCHC7	5947
A	G	0.031416561	5.183123884	3.043427234	PTCH1	469
A	C	0.04822245	6.080645064	-2.85712310	RPL35AP22	3006
A	G	0.04078787	-3.82251022	-2.99997699	RALGPS1	0
A	G	0.04578020	-3.81011885	-2.89191625	LINC00709	194782
T	G	0.030461634	-3.94151076	3.07022159	VCL	0
T	C	0.001368383	5.095948204	-4.88448411	C10orf32-ASI	0
T	G	0.04851855	-3.75147112	2.94620414	YWHAZP5	70268
A	G	0.00015878	5.876393874	-4.47500367	ARNTL	0
T	C	0.014138161	-6.31909471	-3.34042589	PAX6	0
A	G	0.036459165	-4.00937371	2.980796257	SYT13	0
A	G	1.384874524	-6.29636826	-5.28026099	RAPSN	0
T	C	0.01761787	-4.31225213	-3.26490705	GAB2	33
T	C	0.04707712	-3.85633441	2.86844109	CCDC81	0
T	C	3.732943693	6.061807803	-4.76314370	NCAM1	0
T	C	0.034754642	-3.86829282	3.489956044	NTM	0
A	G	0.01540113	-4.23318970	3.552630352	RAD52	0
A	G	0.031151431	-3.92010608	-3.58537049	ANKS1B	0
A	G	0.03833721	-4.50026123	-2.95953870	MVK	25062
T	C	0.048890704	3.695100302	3.50588020	HMGB1	0
A	G	0.04046468	5.30739995	2.936162684	MIR5007	220111
T	C	0.00685473	-4.54888852	-3.65003948	DNAJA1P1	41415
A	G	0.02925657	4.100926082	-3.07316689	RPP40P2	62839
A	G	0.040345897	-4.48204831	-2.93747204	BCRP9	169122
T	C	0.03944742	4.085246753	2.947359847	GPC6	0
A	G	0.00409950	-4.73449609	-3.72373110	SNORD11	143247
T	G	0.00391029	4.75201442	-7.29520711	MAP2K5	0
A	G	0.02974623	-3.94284707	-3.44999352	GLCE	0
A	G	0.00723692	-4.52886988	3.57502083	LINGO1	33139
T	C	0.00657793	-6.32612143	3.57893291	VKORC1	0
A	C	0.00651620	4.568426212	-3.64288433	BAIAP2	0
A	G	0.007496264	4.635543991	-3.54054601	ASXL3	0
T	C	0.02253225	-5.58664370	3.17507069	MIR4318	98879

A	G	0.049791534	4.063897622	2.842081729	DCC	0
T	C	0.006203993	4.586378806	3.630475097	RAB27B	0
A	G	0.000323560	-5.50787070	14.61676158	RPS3AP49	4825
A	G	0.033048758	-3.89258923	5.306581876	PGPEP1	2158
A	C	0.032467436	3.900838719	3.889623173	GATAD2A	0
A	G	0.049934197	-3.68366981	3.799993299	SAE1	0
A	G	0.008294951	5.242517922	-3.50982601	PIGU	0
T	C	0.019901050	-4.12390659	4.275043475	RPSAP64	24010
T	C	0.000858565	7.339246304	-4.10312890	RBX1	48840

func	CADD	RDB	minChrState	PVAL_in_NE	BETA_in_NE	SE_in_NEUR
exonic	19.59	5	4	2.619e-05	0.0099	0.0024
intronic	0.473	2a	5	2.876e-05	-0.0101	0.0024
intronic	0.359	6	4	2.037e-05	0.0101	0.0024
ncRNA_exon	7.283	1f	2	0.0002106	0.0085	0.0023
intronic	11.03	2b	5	8.916e-05	0.0097	0.0025
intergenic	3.262	NA	15	1.333e-05	0.0124	0.0029
intergenic	5.199	NA	14	0.0001437	0.0086	0.0023
ncRNA_intro	8.099	7	15	1.66e-05	0.0098	0.0023
intronic	0.264	5	4	1.752e-10	-0.0183	0.0029
ncRNA_intro	14.92	NA	4	3.578e-07	0.0127	0.0025
intergenic	15.68	6	1	5.717e-05	0.0093	0.0023
intergenic	0.002	NA	2	2.973e-05	-0.0098	0.0023
ncRNA_intro	0.995	3a	1	1.675e-07	-0.0119	0.0023
intronic	5.242	7	4	4.242e-05	0.0153	0.0037
intergenic	5.698	7	14	1.052e-10	-0.0181	0.0028
intronic	18.69	4	1	3.655e-05	-0.01	0.0024
intronic	2.379	6	4	4.271e-05	0.0103	0.0025
intronic	10.84	NA	5	0.0001234	0.013	0.0034
ncRNA_intro	4.365	4	5	9.19e-05	-0.0117	0.003
intergenic	10.59	5	5	1.403e-05	-0.0125	0.0029
ncRNA_intro	7.347	7	5	4.089e-05	-0.0105	0.0026
intronic	1.762	1f	4	2.281e-05	-0.012	0.0028
ncRNA_intro	1.658	5	5	0.0001499	-0.0094	0.0025
intronic	0.994	7	13	5.425e-06	0.0122	0.0027
intergenic	1.06	5	5	0.0001257	-0.0089	0.0023
intergenic	1.677	6	8	0.0001641	0.0091	0.0024
intronic	0.827	NA	1	4.47e-06	-0.0107	0.0023
intronic	0.672	7	7	9.519e-07	-0.0129	0.0026
intronic	0.155	7	5	4.644e-05	-0.0103	0.0025
intronic	2.02	6	5	2.303e-06	-0.0121	0.0026
intronic	6.546	7	5	0.0001223	-0.0088	0.0023
ncRNA_intro	5.553	7	5	8.669e-05	-0.0103	0.0026
intronic	5.438	6	4	0.0001018	0.0101	0.0026
ncRNA_intro	21.7	4	1	4.571e-10	-0.0209	0.0034
intronic	4.719	7	5	4.821e-08	0.0123	0.0023
intronic	2.724	7	5	2.412e-05	-0.0142	0.0034
upstream	2.944	4	1	3.908e-05	-0.0116	0.0028
NA	1.465	7	5	3.719e-06	0.0121	0.0026
ncRNA_intro	6.522	6	9	9.995e-07	0.0111	0.0023
ncRNA_intro	5.718	5	7	8.38e-06	-0.0151	0.0034
intergenic	2.545	4	2	2.976e-06	-0.0107	0.0023
ncRNA_intro	0.072	7	5	1.145e-09	0.0211	0.0035
intergenic	14.32	6	7	1.332e-06	0.011	0.0023

intronic	0.08		5	4	4.529e-05	-0.0105	0.0026
intronic	0.073		7	5	1.878e-05	0.0125	0.0029
intronic	3.211		7	4	1.309e-06	0.0117	0.0024
intergenic	0.722	1f		5	0.0001053	0.0088	0.0023
intergenic	6.493		7	13	1.544e-05	-0.0105	0.0024
ncRNA_intro	17.98	NA		2	4.784e-07	-0.0143	0.0028
intronic	1.235		6	4	2.773e-05	-0.0159	0.0038
intronic	4.097		7	14	2.15e-06	0.0115	0.0024
intergenic	2.738	NA		14	0.0001539	0.0097	0.0026
intronic	6.587	2b		1	1.541e-06	-0.011	0.0023
intergenic	4.858		6	5	1.089e-08	0.0132	0.0023
intergenic	1.015		5	5	0.0001843	-0.0088	0.0024
downstream	8.361		5	4	2.182e-07	0.0215	0.0041
intergenic	0.587	NA		5	1.197e-09	0.0155	0.0025
intronic	2.746		5	5	0.0001321	-0.0097	0.0025
intergenic	1.792		7	9	0.0001389	-0.0087	0.0023
intronic	11.3		4	4	8.097e-05	-0.0133	0.0034
ncRNA_exon	2.402		7	5	3.47e-07	0.0205	0.004
intergenic	11.75		7	5	0.0001758	-0.0088	0.0023
intronic	0.158		7	4	4.193e-09	0.014	0.0024
UTR3	7.915	NA		5	2.631e-10	-0.0177	0.0028
intronic	0.366		5	5	6.088e-05	-0.0125	0.0031
intronic	2.734	1f		4	3.047e-10	-0.0146	0.0023
downstream	2.574	NA		4	1.616e-05	-0.0132	0.0031
intronic	0.262		5	1	0.0001151	-0.0088	0.0023
intronic	3.259		6	5	1.346e-09	0.0139	0.0023
intronic	0.002	NA		5	0.0001096	-0.0088	0.0023
intronic	1.626		6	4	2.304e-05	-0.0098	0.0023
intronic	1.513	NA		9	8.851e-05	-0.0093	0.0024
intergenic	3.665	3a		1	6.787e-06	-0.011	0.0024
UTR3	0.788	NA		4	0.0002198	0.0095	0.0026
intergenic	20.1	NA		9	1.112e-07	0.015	0.0028
intergenic	0.856		7	9	5.393e-06	-0.0109	0.0024
intergenic	3.008		6	5	4.115e-05	0.01	0.0024
intergenic	11.34		5	5	7.393e-06	-0.0126	0.0028
intronic	1.632	NA		5	4.403e-05	0.0095	0.0023
intergenic	1.332		6	9	2.196e-06	-0.011	0.0023
intronic	4.455		6	5	2.014e-06	0.0139	0.0029
intronic	0.391		5	5	8.052e-05	-0.0093	0.0024
intergenic	4.478		7	5	5.93e-06	-0.0106	0.0023
UTR3	3.208	NA		3	2.514e-10	-0.0147	0.0023
intronic	5.168		5	4	4.914e-06	0.0108	0.0024
intronic	3.876		5	1	3.56e-06	0.0105	0.0023
intergenic	5.57		7	5	2.315e-08	-0.0136	0.0024

intronic	0.409		7	9	4.826e-05	0.0092	0.0023
intronic	0.975	3a		5	4.51e-06	0.0125	0.0027
ncRNA_intro	3.068		6	7	3.632e-08	-0.0153	0.0028
intergenic	0.397		6	2	9.918e-05	-0.01	0.0026
intronic	2.765	NA		2	9.586e-05	0.0093	0.0024
intronic	8.72		5	4	0.0002299	-0.0084	0.0023
intronic	12.73	1f		4	1.584e-07	0.0156	0.003
ncRNA_intro	2.645		4	2	3.725e-05	-0.0097	0.0024
intergenic	7.342	3a		1	2.148e-13	0.0184	0.0025

PVAL_in_BM	BETA_in_BM	SE_in_BMI
3.195e-05	-0.0183	0.0044
0.003445	0.0117	0.004
0.0004214	-0.0134	0.0038
1.59e-05	0.0164	0.0038
0.001284	0.0132	0.0041
6.894e-21	-0.0376	0.004
0.0002928	-0.0134	0.0037
7.949e-05	0.0146	0.0037
0.0008793	0.0163	0.0049
4.175e-05	-0.0168	0.0041
0.001227	-0.01	0.0031
3.603e-05	0.0157	0.0038
0.0001863	-0.0142	0.0038
0.003342	0.0179	0.0061
0.001154	0.0156	0.0048
0.0027	-0.012	0.004
0.0006879	0.0115	0.0034
0.002725	-0.0138	0.0046
0.001984	0.0167	0.0054
0.001355	-0.0157	0.0049
0.0001533	-0.0159	0.0042
0.001842	0.0121	0.0039
7.101e-05	0.0133	0.0033
0.0005271	-0.0122	0.0035
1.72e-06	0.0177	0.0037
0.003165	-0.0121	0.0041
5.064e-05	0.0154	0.0038
2.057e-10	-0.0222	0.0035
0.002307	-0.0128	0.0042
3.096e-06	-0.0163	0.0035
0.00247	-0.0112	0.0037
0.001563	0.0136	0.0043
0.003675	-0.0122	0.0042
8.301e-05	0.0175	0.0044
0.003512	-0.0108	0.0037
0.001899	0.0148	0.0048
0.0027	-0.0138	0.0046
0.000746	-0.0145	0.0043
0.0002636	0.0135	0.0037
0.000568	0.0193	0.0056
0.0001863	-0.0142	0.0038
0.0027	0.0174	0.0058
0.002494	0.0092	0.0031

0.001039	-0.0115	0.0035
0.0003132	-0.0173	0.0048
0.002503	-0.0099	0.0033
0.003244	-0.0091	0.0031
0.0002401	-0.012	0.0033
0.0007128	0.0176	0.0052
0.002464	-0.0097	0.0032
0.0003182	-0.0144	0.004
0.00386	0.0099	0.0034
0.000921	0.0105	0.0032
0.0008315	0.0127	0.0038
0.0002817	-0.0138	0.0038
0.002339	0.021	0.0069
0.004275	-0.012	0.0042
0.0027	-0.0129	0.0043
0.003829	-0.0107	0.0037
0.002139	0.0175	0.0057
1.037e-06	-0.0265	0.0054
0.003217	0.0095	0.0032
7.641e-06	-0.0179	0.004
0.0008365	-0.0157	0.0047
0.002875	0.0155	0.0052
1.29e-07	-0.0166	0.0031
0.001095	-0.0136	0.0042
0.004125	0.0109	0.0038
1.906e-06	-0.0181	0.0038
0.0004831	0.0108	0.0031
0.0003814	0.0135	0.0038
0.0003366	-0.0147	0.0041
0.003081	-0.0122	0.0041
0.0004551	0.0127	0.0036
0.003323	0.0138	0.0047
0.0002622	-0.0146	0.004
0.002118	-0.0126	0.0041
0.003309	-0.0141	0.0048
0.003205	0.0112	0.0038
0.0001963	-0.012	0.0032
2.982e-13	-0.0304	0.0042
0.0005606	-0.0138	0.004
0.0003502	0.0143	0.004
0.000345	0.0136	0.0038
0.0002696	-0.0153	0.0042
0.0003993	-0.0131	0.0037
0.001498	0.0127	0.004

0.004482	0.0108	0.0038
0.0002829	0.0167	0.0046
2.196e-48	0.0552	0.0038
1.117e-07	0.0191	0.0036
0.0001004	0.0128	0.0033
0.0001447	0.0152	0.004
0.0004484	-0.0179	0.0051
1.911e-05	0.0171	0.004
4.076e-05	-0.0138	0.0034

locusnum	CHR	LEAD_SNP	LEAD_BP	MinBP	MaxBP	Overlapping
1	1	rs12029004	58118080	58088907	58118080	
2	1	rs1289273	226909233	226903535	226927457	
3	2	rs736699	26913930	26910556	26932887	BMI
4	2	rs6738482	61494901	61242410	61837947	HDL
5	2	rs35942385	144208523	144145478	144272229	
6	2	rs17741344	148557573	148457576	148853296	WHR
7	2	rs13401963	156060402	155654757	156114451	
8	3	rs2606731	11291615	11248656	11557126	
9	3	rs13082065	35731993	35565537	35777848	
10	3	rs13082158	48372470	48172687	48379787	
11	3	rs2282751	50291785	50184538	50412945	TC
12	4	rs12505933	32543816	32487731	32591496	
13	4	rs6858227	37973722	37973722	38007099	
14	4	rs17516389	119239267	118976252	119264162	CAD,T2D
15	4	rs7691325	143879892	143786837	144215346	BMI
16	4	rs13126083	156437388	156420605	156443314	
17	5	rs359477	173305432	173305432	173305432	
18	6	rs1077393	31610529	30997692	32189481	CAD,HDL,LDL,
19	6	rs2396004	43355851	43262303	43364494	T2D
20	6	rs1548291	78835883	78669791	78955109	
21	6	rs12196322	83691384	83615698	83869103	
22	6	rs6925689	126865884	126623947	127080700	CAD,WHR,CR
23	6	rs728035	143178021	143146424	143206826	
24	7	rs2010309	73967401	73907471	73971120	
25	8	rs7002383	8800760	8088230	12199830	CRP
26	8	rs2942202	23418444	23418122	23418444	
27	8	rs4599845	116593598	116464988	116645056	HDL,BMI,LDL
28	8	rs13282237	143326191	143309504	143352206	
29	9	rs7851242	4147764	4103560	4148596	
30	10	rs1926221	9772607	9651373	9963730	
31	10	rs10430801	13490742	13479684	13558641	T2D
32	10	rs77335224	104636276	104565199	105059896	CAD
33	11	rs12281432	10257201	10033265	10370675	
34	11	rs12801635	16352545	16255448	16373664	
35	11	rs12292911	47449072	46331362	48014868	HDL,LDL,TC,T
36	14	rs8004084	75220107	75144618	75377692	HDL,TC
37	15	rs17514846	91416550	91412850	91429042	CAD
38	16	rs77997837	4944256	4817296	4960254	
39	16	rs1549299	31154146	30916129	31155458	BMI,TG
40	16	rs74613454	89895469	89715375	90010172	
41	17	rs6502647	18224977	18104433	18271876	
42	17	rs55938136	43798360	43798360	43798360	CAD
43	18	rs9807547	48727203	48679989	48779767	

44	20	rs1569832	10828300	10819210	10862433
45	21	rs12329923	18958681	18958316	19009100
46	21	rs7279867	43468984	43468984	43468984

A1	A2	FDR	Z_in_NEUR	Z_in_DBP	nearestGene	dist
A	G	0.038356234	-3.89792612	-3.59619966	DAB1	0
A	G	0.035692606	-3.79024296	3.95138564	ITPKB	0
A	G	0.012048775	4.189637856	8.817064082	KCNK3	1688
A	G	0.006263684	4.453605976	-4.26985510	USP34	0
T	G	0.000580405	-5.11062192	-4.92082537	ARHGAP15	0
T	C	0.010959481	5.543254995	4.080960382	ACVR2A	44512
A	G	0.007866557	4.332389048	-4.25334577	MTND2P20	60272
A	C	0.043049663	-3.71388694	-4.42302161	HRH1	0
T	C	0.003615495	5.793852205	-4.43899479	ARPP21	0
T	C	0.046229554	3.684669045	5.318893884	SPINK8	2638
A	G	0.014856009	4.116661500	4.509045314	GNAI2	0
T	C	0.045845478	3.688026632	3.538111396	RP11-240A16	188973
T	C	0.028864671	-4.14622614	-3.71240392	TBC1D1	0
T	C	0.026070249	-4.33016153	3.752471132	PRSS12	0
A	C	0.023363590	-4.12150824	3.796421193	RP11-284M1	0
T	C	0.036202227	-4.06423623	-3.61895068	MTND1P22	52412
T	C	0.041248764	-3.73208685	3.612550529	CPEB4	9850
A	G	7.522696138	-5.66008277	-5.36428610	BAG6	0
A	G	0.041562395	3.728922864	4.122489315	ZNF318	18634
A	C	0.045599587	3.690174809	3.712527200	SNORD112	188894
A	G	0.016797886	4.460286922	-3.92364287	UBE3D	0
T	C	0.002866985	4.683222269	-4.50713121	RNU6-200P	45548
A	G	0.039632761	3.748352456	3.730104986	HIVEP2	0
T	C	0.038189028	-3.76283209	-3.60872970	GTF2IRD1	0
T	G	7.780360251	-5.74092641	5.358039177	SNORA70	18324
A	C	0.033405213	3.816788442	-4.07684959	SLC25A37	0
T	C	0.006184858	-4.40951998	-4.44483340	TRPS1	0
A	G	0.029699740	3.932262793	3.700588552	TSNARE1	0
A	G	0.006086832	-5.54491321	-4.27904134	GLIS3	0
T	C	0.025625241	3.918369428	3.769919822	HSP90AB7P	6227
A	G	0.015178024	-4.66487812	3.962400286	BEND7	0
T	C	0.000631086	-5.23859919	-4.90001364	C10orf32-ASN	00:00
A	G	0.018673347	4.102958747	3.883650003	SBF2	0
T	C	0.037118421	3.773613449	-5.74220787	SOX6	0
A	G	4.505386602	6.222434231	5.933579707	PSMC3	1078
T	C	0.008002775	-6.54353852	-4.18980035	YLPM1	9961
A	C	0.005662919	-4.43830518	5.971556949	FURIN	0
T	C	0.022030495	-3.97432207	-4.62105148	PPL	0
A	G	0.046199109	4.476124980	-3.51880206	PRSS36	0
T	G	0.041854523	3.787452123	3.558978502	SPIRE2	0
A	G	0.009704679	-4.26300057	-4.18386051	SMCR8	0
A	G	0.009075446	-11.5419461	4.146768972	CRHR1:RP11-	00:00
A	G	0.046172457	-3.78485212	3.519046983	MEX3C	0

A	G	0.010853815[illegible]	4.231536809[illegible]	4.084351929	RP4-697P8.3	27680
A	G	0.039868812[illegible]	-4.20689793[illegible]	3.580147736	CXADR	0
T	C	0.045560572[illegible]	3.690515557[illegible]	3.743050049	UMODL1	14083
			18			
			28			

func	CADD	RDB	minChrState	posMapFilt	eqtlMapFilt	ciMapFilt
intronic	2.97	5	9	1	0	0
intronic	2.307	5	4	1	0	0
intergenic	0.475	5	5	1	0	0
intronic	2.003	6	4	1	1	0
ncRNA_intror	18.94	5	4	1	0	0
intergenic	3.268	7	5	0	1	0
intergenic	0.847	7	9	0	0	0
intronic	0.111	NA	4	1	1	0
intronic	8.987	5	4	1	0	0
intergenic	0.158	5	5	1	1	0
intronic	0.893	1f	3	1	1	0
intergenic	4.657	6	9	0	0	0
intronic	7.106	7	5	1	1	0
intronic	8.097	7	4	1	0	0
ncRNA_intror	3.157	5	5	0	1	0
ncRNA_intror	1.44	6	14	0	0	0
intergenic	9.06	1f	2	1	0	0
intronic	5.552	5	4	1	1	0
intergenic	2.553	6	5	0	0	0
intergenic	0.003	NA	15	0	0	0
intronic	3.536	5	5	1	1	0
intergenic	2.754	7	5	0	0	0
intronic	7.163	4	1	1	0	1
intronic	3.881	NA	4	1	0	0
intergenic	0.02	6	5	0	1	0
intronic	5.229	NA	2	1	1	0
intronic	3.683	7	5	1	1	0
intronic	5.521	5	9	1	1	0
intronic	10.55	4	1	1	0	0
intergenic	3.393	NA	9	0	0	0
intronic	5.773	6	4	1	1	0
intronic	3.704	7	4	1	1	0
intronic	2.925	7	5	1	0	0
intronic	10.29	6	4	1	0	0
intergenic	4.383	6	1	1	1	0
intergenic	2.999	7	7	1	1	0
intronic	10.53	4	1	1	1	0
intronic	0.333	5	4	1	1	0
exonic	14.24	NA	1	1	1	0
intronic	8.603	4	1	1	1	0
intronic	4.359	1f	4	1	1	0
ncRNA_intror	3.353	6	5	1	1	0
intergenic	2.261	6	5	1	0	0

ncRNA_intror	1.861	7	5	0	0	0
intronic	1.696	6	5	1	0	0
intergenic	1.131	7	5	0	0	0

PVAL_in_NEU	BETA_in_NEU	SE_in_NEUR	PVAL_in_DBP	BETA_in_DBP
9.702e-05	-0.0089	0.0023	0.0003229	-0.1028
0.0001505	-0.0086	0.0023	7.77e-05	0.1165
2.794e-05	0.0098	0.0023	1.175e-18	0.259
8.444e-06	0.0119	0.0027	1.956e-05	-0.1346
3.211e-07	-0.012	0.0023	8.618e-07	-0.1477
2.969e-08	0.0157	0.0028	4.485e-05	0.1526
1.475e-05	0.0106	0.0025	2.106e-05	-0.1317
0.0002041	-0.0089	0.0024	9.733e-06	-0.129
6.879e-09	0.0133	0.0023	9.038e-06	-0.1261
0.000229	0.169	0.0459	1.044e-07	0.1686
3.844e-05	0.0142	0.0034	6.512e-06	0.2916
0.000226	0.0146	0.0039	0.000403	0.1771
3.38e-05	-0.0106	0.0026	0.0002053	-0.1195
1.49e-05	-0.0168	0.0039	0.0001751	0.2004
3.764e-05	-0.0109	0.0026	0.0001468	0.1169
4.819e-05	-0.0097	0.0024	0.0002958	-0.1052
0.0001899	-0.0085	0.0023	0.0003032	0.104
1.513e-08	-0.0128	0.0023	8.127e-08	-0.1462
0.0001923	0.0085	0.0023	3.748e-05	0.1252
0.0002241	0.0087	0.0024	0.0002052	0.2256
8.185e-06	0.0152	0.0034	8.722e-05	-0.1765
2.824e-06	0.0106	0.0023	6.571e-06	-0.1341
0.000178	0.0093	0.0025	0.0001914	0.1214
0.000168	-0.0104	0.0028	0.0003077	-0.1221
9.416e-09	-0.2855	0.0497	8.413e-08	0.1617
0.0001352	0.0087	0.0023	4.565e-05	-0.1148
1.036e-05	-0.0101	0.0023	8.796e-06	-0.1239
8.415e-05	0.009	0.0023	0.0002151	0.1049
2.941e-08	-0.0142	0.0026	1.877e-05	-0.1326
8.915e-05	0.0102	0.0026	0.0001633	0.1178
3.088e-06	-0.0115	0.0025	7.42e-05	0.1205
1.618e-07	-0.0212	0.004	9.583e-07	-0.2209
4.079e-05	0.0093	0.0023	0.0001029	0.1068
0.0001609	0.0104	0.0028	9.345e-09	-0.2133
4.895e-10	0.0144	0.0023	2.964e-09	0.1767
6.008e-11	-0.0157	0.0024	2.792e-05	-0.1257
9.067e-06	-0.0102	0.0023	2.35e-09	0.1752
7.058e-05	-0.0109	0.0027	3.818e-06	-0.1564
7.601e-06	0.0111	0.0025	0.0004335	-0.1168
0.0001522	0.0141	0.0037	0.0003723	0.2144
2.017e-05	-0.0104	0.0024	2.866e-05	-0.14
8.107e-31	-0.0312	0.0027	3.372e-05	0.4091
0.0001538	-0.161	0.0425	0.0004331	0.1009

2.321e-05	0.0101	0.0024	4.42e-05	0.1188
2.589e-05	-0.0099	0.0023	0.0003434	0.1031
0.0002238	0.009	0.0024	0.0001818	0.1103

locusnum	CHR	LEAD_SNP	LEAD_BP	MinBP	MaxBP	Overlapping E
1	1	rs3795310	8431607	8390051	8892577	WHR
2	1	rs4650199	73652122	73307627	73991792	
3	1	rs59435160	210030730	209948711	210398565	
4	2	rs736699	26913930	26911509	26932796	BMI
5	2	rs7581001	43914278	43909878	43931585	
6	2	rs62132359	44977657	44905806	45004016	
7	2	rs848286	58394543	57931347	58505679	BMI
8	2	rs2421116	61488411	61349446	61837947	
9	2	rs13409451	144257639	144145478	144272229	
10	2	rs1867648	155619314	155614280	155631428	
11	2	rs10165846	176727834	176717741	176735925	
12	3	rs13082065	35731993	35644566	35777848	
13	3	rs1989839	50378946	50184538	50420554	TC
14	3	rs12637791	85525323	84964976	85789703	BMI
15	4	rs10084926	37996827	37973722	38008202	
16	4	rs16854051	42123892	41977902	42161491	
17	4	rs11722027	144050017	144028173	144215346	BMI
18	4	rs13126083	156437388	156420605	156443314	CAD
19	5	rs140323342	122075342	122005153	122380190	
20	5	rs216139	149440723	149341038	149445921	
21	6	rs2269426	32076499	31578772	32189481	HDL,CRP,LDL,
22	6	rs10948071	43280713	43260660	43397259	DBP, PP
23	6	rs2243758	51436530	51415907	51451600	
24	6	rs1490384	126851160	126659043	127080700	CAD,WHR,CR
25	7	rs58673065	1885600	1843200	2110850	CAD
26	7	rs6460902	12255511	12200060	12285140	CAD
27	7	rs9647718	73939393	73907471	73971120	
28	7	rs7784849	117611250	117599514	117636111	
29	7	rs1541481	139465277	139404666	139491967	
30	8	rs2736313	11086942	8088230	12199830	CRP
31	8	rs3134112	110104855	110058677	110106809	
32	8	rs10098073	143309504	143308772	143353512	
33	9	rs7870079	96161300	96155812	96381916	
34	9	rs359590	127977545	127766897	128008537	
35	10	rs10906382	13516426	13479684	13611368	T2D
36	10	rs67214975	18727251	18681659	18727251	
37	10	rs11000909	75944828	75867193	76421529	CAD
38	10	rs77335224	104636276	104487871	105059896	CAD, BMI
39	11	rs1988724	10239723	9958403	10370675	HDL
40	11	rs12801635	16352545	16255448	16373664	
41	11	rs10835353	28547534	28379460	28577867	
42	11	rs11038371	45282531	45258966	45345244	BMI
43	11	rs7107356	47676170	47175327	49128599	HDL,BMI,CRP

44	11	rs7125689	58168209	57993924	58174167	
45	11	rs990706	78135704	77909014	78271614	CAD,BMI,LDL,
46	11	rs74613479	113577200	113577200	113873852	
47	12	rs11609027	49801156	49737114	50160662	
48	12	rs1245811	79637453	79552449	79713434	
49	13	rs7338530	80051850	79855297	80166707	
50	14	rs10133241	75098193	75062398	75113682	TC
51	14	rs112635299	94838142	94838142	94844947	
52	15	rs7176782	69477380	69415482	69569464	BMI
53	15	rs17514846	91416550	91412850	91429042	CAD
54	16	rs79338890	4905127	4817296	4960254	
55	17	rs10852923	2086355	2078890	2090341	
56	17	rs3027178	8053085	8036564	8164352	
57	17	rs12936769	18189625	18135401	18271522	
58	17	rs2165846	44941366	44941366	44947821	
59	18	rs584564	53227648	53198836	53413423	
60	20	rs6040195	10831812	10819210	10862433	

A1	A2	FDR	Z_in_NEUR	Z_in_SBP	nearestGene	dist
T	C	0.000668883	5.269360319	-5.07139988	RERE	0
T	C	0.042290573	-3.78761547	-3.77099182	RN7SKP19	5164
A	C	0.049511197	-3.71914282	3.764025234	DIEXF	0
A	G	0.015296420	4.189637856	12.34041840	KCNK3	1688
T	C	0.024046839	4.026056772	3.766729559	PLEKHH2	0
T	G	0.042271533	3.807462536	-3.54061210	CAMKMT	0
T	C	0.001609565	5.452308867	-4.62181345	FANCL	0
A	G	0.037076668	4.512990122	-3.59515379	USP34	0
A	G	0.022447536	4.795462414	3.793899028	ARHGAP15	0
T	C	0.033587453	-3.88459644	4.019749719	KCNJ3	0
T	C	0.047938136	4.237522278	-3.48676541	EXTL2P1	19803
T	C	0.006193812	5.793852205	-4.23662957	ARPP21	0
A	G	0.040461981	-5.10311943	-3.55799248	ZMYND10-AS	00:00
T	G	0.004534565	-5.00576709	-4.33164415	CADM2	0
T	C	0.007474501	-4.46847287	-4.17760089	TBC1D1	0
T	C	0.006918047	-5.43567296	4.202143014	BEND4	0
A	G	0.028960675	3.944789826	-5.83441381	RP11-284M1	0
T	C	0.023724333	-4.06423623	-3.77222230	MTND1P22	52412
T	G	0.008924987	-4.38629070	-4.47035464	RP11-166A12	8961
A	G	0.044139870	-3.76869995	-4.02847428	CSF1R	0
A	G	0.000103104	7.854646885	5.295794567	TNXB:ATF6B	00:00
T	C	0.020417707	-4.08127148	-6.82311061	ZNF318	0
A	C	0.040571794	4.048758690	-3.55693986	PKHD1	43567
T	C	0.001090772	-5.25379094	4.723868566	MIR588	45300
A	G	0.007608568	4.443784843	4.283833989	MAD1L1	0
A	G	0.028238536	5.923062850	3.704150873	TMEM106B	0
T	C	0.017570317	4.138161442	3.891077615	GTF2IRD1	0
A	G	0.018563612	-4.45649639	-3.86519605	CTTNBP2	97056
T	C	0.012330550	-4.26905825	-4.15527255	HIPK2	0
T	C	7.400049659	-5.84975419	5.495185468	LINC00529	18192
A	G	0.018658799	4.114809086	3.890980396	TRHR	0
A	C	0.002473825	4.843923441	4.689480151	TSNARE1	0
T	C	0.022378200	-4.27031166	3.795073035	RNU6-829P	23350
A	G	0.006987383	-4.51173332	4.198828009	RABEPK	0
T	C	0.009473172	-4.36400954	4.352327249	BEND7	0
A	C	0.017683057	-4.13574818	-4.80084186	CACNB2	0
A	G	0.024853930	4.005333665	-3.96424047	ADK	0
T	C	0.000739734	-5.23859919	-11.6737003	C10orf32-ASN	00:00
A	C	0.004048737	4.669931627	4.815679258	SBF2	0
T	C	0.043612573	3.773613449	-4.93512364	SOX6	0
A	G	0.035291391	3.864098991	-3.61668506	METTL15	192479
A	G	0.023673621	4.024073715	-4.12984999	SYT13	0
A	G	3.077639126	-7.04747006	-6.46799771	AGBL2	4972

A	G	0.014999760	-4.197061938	-4.303527462	OR5B3	1727
T	C	0.022604796	-4.177137955	-3.791235056	RP11-452H21	0
T	G	0.048617013	4.512460074	3.481078020	TMPRSS5	104
T	C	0.002997058	-4.776356304	4.548888525	SPATS2	0
T	C	0.047231174	3.882236835	3.492679544	SYT1	0
T	C	0.029101524	3.942847079	-3.892125051	NDFIP2-AS1	0
A	G	0.005325705	-4.571952712	-4.368504919	LTBP2	18886
T	G	0.045356159	-3.757528249	-3.606796994	SERPINA1	4941
A	G	0.032700324	-3.942847079	-3.645955574	GLCE	0
A	C	0.007723066	-4.438305181	7.840208204	FURIN	0
T	G	0.021285192	4.065303482	4.020154446	UBN1	0
T	G	0.017614351	4.722206172	3.883650003	SMG6	0
T	G	0.014713839	4.204206208	4.102448990	PER1	0
T	G	0.043035830	3.779727639	5.456321301	TOP3A	0
A	G	0.016492244	4.773223944	3.908554507	WNT9B	0
A	G	0.009061258	-4.665278051	4.115405097	TCF4	0
A	G	0.022789379	4.055626981	3.788106142	RP4-697P8.3	24168
			24			
			36			

func	CADD	RDB	minChrState	posMapFilt	eqtlMapFilt	ciMapFilt
intronic	0.722	4	2	1	1	0
intergenic	2.613	6	5	0	0	0
UTR3	2.036	5	4	1	1	0
intergenic	0.475	5	5	1	0	0
intronic	1.835	7	4	1	1	0
intronic	0.083	6	5	1	0	0
intronic	1.87	NA	4	1	0	0
intronic	4.877	6	4	1	1	0
ncRNA_intror	1.167	6	5	1	0	0
intronic	4.495	3a	9	1	0	0
intergenic	2.386	7	7	0	0	0
intronic	8.987	5	4	1	0	0
ncRNA_intror	11.3	NA	1	1	1	1
intronic	11.94	6	5	1	0	0
intronic	0.193	5	2	1	1	0
intronic	0.06	7	4	1	1	0
ncRNA_intror	2.192	7	14	0	1	0
ncRNA_intror	1.44	6	14	0	0	0
intergenic	2.117	6	5	0	0	0
intronic	4.054	5	4	1	1	0
intronic	0.475	NA	1	1	1	0
intronic	1.805	2c	2	1	0	0
intergenic	2.365	7	9	0	0	0
intergenic	18.6	7	5	0	0	0
intronic	4.431	5	2	1	0	0
intronic	0.71	6	4	1	0	0
intronic	1.117	7	4	1	0	0
intergenic	5.97	6	14	0	0	0
intronic	6.611	NA	4	1	0	0
intergenic	1.004	1f	5	0	1	0
intronic	3.241	6	9	1	0	0
intronic	3.18	4	5	1	1	0
intergenic	0.057	7	6	0	0	0
intronic	8.289	6	4	1	1	0
intronic	3.595	7	4	1	0	0
intronic	0.018	5	5	1	0	0
intronic	9.324	5	5	1	1	0
intronic	3.704	7	4	1	1	0
intronic	3.537	NA	5	1	1	0
intronic	10.29	6	4	1	0	0
ncRNA_intror	1.339	7	5	0	0	0
intronic	0.436	6	5	1	1	0
intergenic	3.079	6	13	1	1	0

intergenic	1.437		7	14	1	0	0
ncRNA_exoni	1.788	2b		2	1	1	0
upstream	16.03	2b		1	1	0	0
intronic	10.38		7	4	1	0	0
intronic	7.901		5	5	1	0	0
ncRNA_intror	7.374		6	2	1	1	0
intergenic	7.56		5	2	0	1	0
intergenic	0.272		5	5	1	0	0
intronic	0.391		5	5	1	1	0
intronic	10.53		4	1	1	1	0
intronic	5.081		7	4	1	1	0
intronic	3.092		5	4	1	1	0
exonic	1.193	NA		3	1	1	0
intronic	1.922		7	4	1	1	0
intronic	12.21	1f		5	1	1	0
intronic	8.736		5	4	1	0	0
ncRNA_intror	7.397		7	5	0	0	0

PVAL_in_NEU	BETA_in_NEU	SE_in_NEUR	PVAL_in_SBP	BETA_in_SBP
1.369e-07	0.012	0.0023	3.949e-07	-0.194
0.0001521	-0.0086	0.0023	0.0001626	-0.1425
0.0001999	-0.0113	0.003	0.0001672	0.1871
2.794e-05	0.0098	0.0023	5.487e-35	0.4862
5.672e-05	0.0092	0.0023	0.0001654	0.1424
0.0001404	0.0116	0.0031	0.0003992	-0.1852
4.972e-08	0.0128	0.0024	3.804e-06	-0.1852
6.392e-06	0.0121	0.0027	0.0003242	-0.1516
1.623e-06	0.0112	0.0023	0.0001483	0.1543
0.0001025	-0.0088	0.0023	5.826e-05	0.1463
2.26e-05	0.0096	0.0023	0.0004889	-0.128
6.879e-09	0.0133	0.0023	2.269e-05	-0.1612
3.341e-07	-0.0182	0.0036	0.0003737	-0.1946
5.564e-07	-0.0118	0.0024	1.48e-05	-0.1653
7.878e-06	-0.0117	0.0026	2.946e-05	-0.1741
5.459e-08	-0.0155	0.0028	2.644e-05	0.1967
7.987e-05	0.0091	0.0023	5.398e-09	-0.218
4.819e-05	-0.0097	0.0024	0.0001618	-0.1466
1.153e-05	-0.3041	0.0693	7.809e-06	-0.2531
0.0001641	-0.0087	0.0023	5.614e-05	-0.1714
4.009e-15	0.0185	0.0024	1.185e-07	0.2003
4.479e-05	-0.0095	0.0023	8.909e-12	-0.2755
5.149e-05	0.0092	0.0023	0.0003752	-0.1324
1.49e-07	-0.0119	0.0023	2.314e-06	0.1845
8.839e-06	0.012	0.0027	1.837e-05	0.1792
3.16e-09	0.0136	0.0023	0.0002121	0.1392
3.501e-05	0.0116	0.0028	9.98e-05	0.1886
8.331e-06	-0.0103	0.0023	0.000111	-0.1472
1.963e-05	-0.0124	0.0029	3.249e-05	-0.2068
4.923e-09	-0.2579	0.0441	3.903e-08	0.2324
3.875e-05	0.0095	0.0023	9.984e-05	0.1468
1.273e-06	0.0111	0.0023	2.739e-06	0.1832
1.952e-05	-0.0098	0.0023	0.0001476	0.1516
6.43e-06	-0.0103	0.0023	2.683e-05	0.1567
1.277e-05	-0.0108	0.0025	1.347e-05	0.1856
3.538e-05	-0.0094	0.0023	1.58e-06	-0.1848
6.193e-05	0.0135	0.0034	7.363e-05	-0.2106
1.618e-07	-0.0212	0.004	1.737e-31	-0.7031
3.013e-06	0.0106	0.0023	1.467e-06	0.1856
0.0001609	0.0104	0.0028	8.01e-07	-0.2458
0.0001115	0.0088	0.0023	0.0002984	-0.1372
5.72e-05	0.0092	0.0023	3.63e-05	-0.1541
1.822e-12	-0.0159	0.0023	9.931e-11	-0.237

2.704e-05	-0.0098	0.0023	1.681e-05	-0.1641
2.952e-05	-0.0127	0.003	0.0001499	-0.1754
6.408e-06	0.0303	0.0067	0.0004994	0.4772
1.785e-06	-0.0213	0.0044	5.393e-06	0.2972
0.0001035	0.0091	0.0024	0.0004782	0.1515
8.052e-05	0.0091	0.0023	9.937e-05	-0.1566
4.832e-06	-0.0105	0.0023	1.251e-05	-0.1702
0.0001716	-0.0298	0.0079	0.00031	-0.6084
8.052e-05	-0.0093	0.0024	0.0002664	-0.1599
9.067e-06	-0.0102	0.0023	4.498e-15	0.3087
4.797e-05	0.0096	0.0024	5.816e-05	0.1574
2.333e-06	0.0107	0.0023	0.0001029	0.1479
2.62e-05	0.0106	0.0025	4.088e-05	0.1901
0.000157	0.0092	0.0024	4.861e-08	0.2219
1.813e-06	0.0114	0.0024	9.285e-05	0.1541
3.082e-06	-0.0191	0.0041	3.865e-05	0.3232
5,00E-05	0.0097	0.0024	0.0001518	0.1496

locusnum	CHR	LEAD_SNP	LEAD_BP	MinBP	MaxBP	Overlapping
1	1	rs11121210	8708529	8390051	8892577	PP
2	1	rs1460940	72814617	72628347	72959039	BMI,TG,T2D
3	1	rs4442420	174168805	174050667	174921365	
4	2	rs12612492	24093756	23890084	24257682	CAD
5	2	rs35720761	43519977	43449385	43850357	T2D
6	2	rs13387871	58737900	58683715	58822464	HDL
7	2	rs11685710	148892754	148496105	148955901	DBP
8	3	rs2648347	12057200	12029463	12307143	
9	3	rs9310510	16877206	16848835	16879840	
10	3	rs12489828	52567014	52540773	52847601	CAD,BMI,HD
11	5	rs16903232	87831734	87782483	87919700	BMI
12	6	rs3129953	32361821	29659255	32946837	TG,DBP,PP
13	6	rs6907898	126666416	126657472	127080700	CAD,CRP,T2D
14	7	rs6466484	114012911	113865735	114192545	
15	7	rs727164	117532152	117494829	117594838	
16	9	rs992548	23787897	23747791	23822506	PP
17	11	rs4938021	113364803	113317745	113413565	
18	15	rs4776361	67762056	67680209	68140315	BMI
19	17	rs2435200	44071851	44065263	44078982	CAD,HDL
20	18	rs17700144	57811982	57728947	57913434	CAD,BMI,HD
21	19	rs9636202	18449238	18449238	18474892	BMI
22	22	rs17822045	47208009	47161507	47234866	

A1	A2	FDR	Z_in_NEUR	Z_in_WHR	nearestGene	dist
T	C	0.019959133	4.260796112	-3.99026325	RERE	0
A	G	0.01563222C	4.354616623	-4.45218722	RPL31P12	47104
T	G	0.013872331	-4.77953624	3.73195426S	RABGAP1L	0
T	C	0.03115546C	4.079101993	3.706672763	ATAD2B	0
T	C	0.034309837	4.12991334C	3.46511743C	THADA	0
T	C	0.005626781	-5.19510893	3.959839985	LINC01122	0
A	G	0.039489578	-6.92216268	3.418358785	MBD5	0
T	G	0.03459312S	4.035526456	3.567793525	SYN2	0
A	G	0.025941271	-5.20139331	3.553597714	PLCL2	0
T	G	0.019768804	-4.41310190	3.63313355C	NT5DC2	0
T	C	0.048125027	3.975335868	3.349354795	LINC00461	0
T	C	0.001484584	-5.15890371	4.360105083	BTNL2	0
T	C	0.00894680S	-5.38811623	3.846126144	CENPW	0
A	C	0.004706376	-4.78877798	4.264890793	FOXP2	0
A	G	0.029974515	4.297160416	3.508878751	CTTNBP2	17958
A	G	0.045397531	-4.50693748	3.370617184	ELAVL2	0
T	C	0.00968111C	8.054752179	3.826458602	DRD2	18389
A	G	0.019451087	4.270540276	3.903019616	IQCH:IQCH-A	00:00
A	G	0.016545455	6.934937776	3.68355901S	MAPT	0
A	G	0.000430242	-5.50787070	5.892672096	RPS3AP49	4825
A	G	0.047697003	-3.89258923	4.677778185	PGPEP1	2158
T	C	0.028151452	4.122059608	3.706672763	TBC1D22A	0

func	CADD	RDB	minChrState	PVAL_in_NE	BETA_in_NE	SE_in_NEUR
intronic	0.359	6	4	2.037e-05	0.0101	0.0024
intergenic	3.262	NA	15	1.333e-05	0.0124	0.0029
intronic	0.552	6	5	1.757e-06	-0.0111	0.0023
intronic	3.388	1f	4	4.521e-05	0.0137	0.0034
exonic	22.4	NA	4	3.629e-05	0.014	0.0034
ncRNA_intro	3.509	6	5	2.046e-07	-0.0151	0.0029
intronic	8.232	6	5	4.448e-12	-0.0159	0.0023
ncRNA_intro	3.273	7	5	5.448e-05	0.0114	0.0028
NA	6.375	5	5	1.978e-07	-0.0128	0.0025
intronic	6.779	1f	1	1.019e-05	-0.01	0.0023
ncRNA_intro	0.89	7	5	7.028e-05	0.0158	0.004
exonic	4.039	1b	2	2.484e-07	-0.0156	0.003
intronic	5.277	6	5	7.12e-08	-0.0123	0.0023
intronic	5.089	3a	5	1.678e-06	-0.0112	0.0023
intergenic	0.047	5	5	1.73e-05	0.0105	0.0025
intronic	2.787	7	5	6.577e-06	-0.0122	0.0027
intergenic	0.724	5	5	7.964e-16	0.0188	0.0023
ncRNA_intro	0.743	6	5	1.95e-05	0.011	0.0026
intronic	0.054	6	4	4.064e-12	0.0159	0.0023
ncRNA_intro	3.068	6	7	3.632e-08	-0.0153	0.0028
intergenic	0.397	6	2	9.918e-05	-0.01	0.0026
intronic	13.29	5	4	3.755e-05	0.0104	0.0025

PVAL_in_WH	BETA_in_WH	SE_in_WHR
6.6e-05	-0.017	0.0043
8.5e-06	-0.02	0.0045
0.00019	0.013	0.0034
0.00021	0.019	0.005
0.00053	0.032	0.0091
7.5e-05	0.021	0.0053
0.00063	0.012	0.0034
0.00036	0.015	0.0042
0.00038	0.016	0.0045
0.00028	0.012	0.0034
0.00081	0.019	0.0056
1.3e-05	0.021	0.0047
0.00012	0.014	0.0037
2,00E-05	0.018	0.0043
0.00045	0.015	0.0044
0.00075	0.013	0.004
0.00013	0.013	0.0035
9.5e-05	0.015	0.0039
0.00023	0.013	0.0034
3.8e-09	0.024	0.0041
2.9e-06	0.019	0.004
0.00021	0.017	0.0046

locusnum	CHR	LEAD_SNP	LEAD_BP	MinBP	MaxBP	Overlapping
1	1	rs7537621	230236401	230230169	230261929	
2	2	rs2075171	58688907	58617313	58846099	WHR
3	2	rs935862	61479131	61369787	61763207	SBP,DBP
4	3	rs9853056	52555957	52540773	52847601	TC,LDL,PP
5	3	rs3849570	81792112	81573660	81982771	CAD,BMI
6	3	rs3732359	119536429	119533346	119816106	
7	3	rs1153877	136004925	135814009	136508008	CAD,BMI,TG,
8	5	rs7724884	107598962	107282893	107908296	CAD,BMI
9	6	rs415929	32189032	32011235	32681631	CAD,BMI,LDL
10	6	rs3756784	131950233	131897278	131973932	
11	6	rs1159327	152048022	152027074	152052043	
12	8	rs9886524	103533827	103530644	103537024	
13	8	rs2737219	116635688	116409058	116666712	BMI,LDL,DBP
14	9	rs10989112	103252812	103173978	103252812	
15	11	rs11042717	10303939	9961079	10369340	SBP
16	11	rs10832027	13357183	13354509	13370535	CAD,BMI,TG,
17	11	rs12419692	47624714	47372377	47946836	BMI,TC,T2D,
18	12	rs11067376	110027795	109774263	110069478	BMI
19	13	rs11147458	31823239	31703734	31839274	
20	14	rs12588415	75278211	75120628	75378185	TC,LDL,DBP
21	16	rs17139608	6281175	6266886	6285091	
22	17	rs11078936	38197914	38128181	38219005	TC,LDL
23	17	rs2016730	44204373	44040184	44833217	CAD,WHR
24	18	rs17700144	57811982	57728947	57912226	CAD,BMI,TG,
25	19	rs10409835	32888660	32830261	32994338	TG
26	20	rs3092169	44702086	44674283	44729089	
27	20	rs6021721	50746651	50734238	50771635	
28	22	rs5763767	30493882	30223888	30592487	
29	22	rs138457	38898051	38869463	39152412	TG,CRP

A1	A2	FDR	Z_in_NEUR	Z_in_HDL	nearestGene	dist
T	C	0.012572645	4.782649071	3.595475180	GALNT2	0
A	G	0.038472472	4.089448530	3.374276454	LINC01122	0
A	G	0.046938224	4.087205693	3.207057906	USP34	0
T	C	0.031968236	4.167839738	3.444413369	STAB1	0
A	C	0.042443843	-4.27951620	3.240787512	GBE1	0
A	G	0.035952675	4.122981343	3.293324872	NR1I2	0
T	C	0.009464887	-6.96768439	3.669947804	PCCB	0
T	C	0.021374541	4.337824476	3.747367749	FBXL17	0
T	C	0.001072081	-5.42821640	4.496688470	NOTCH4	0
T	G	0.036480763	4.112852555	3.311069868	ENPP3	0
T	C	0.006051502	4.845175200	4.164160673	ESR1	0
T	C	0.037935033	4.738317485	3.275969317	KB-1980E6.3	7140
A	G	0.001616985	5.303987619	7.207305190	TRPS1	0
T	C	0.031979739	-4.61148630	3.328452167	MSANTD3-TI	00:00
T	C	0.005295251	-4.89527170	4.318771673	SBF2	0
A	G	0.001052755	-5.43398246	4.960947919	ARNTL	0
A	C	6.115331432	-6.15346105	9.748236740	C1QTNF4	8502
A	G	1.441318836	-7.34595179	6.542967352	MVK	0
A	G	0.022974071	-4.30644387	3.760166949	B3GALTL	0
A	G	0.036027356	6.863083093	3.292672497	YLPM1	0
T	C	0.044862523	-4.12527005	3.222674009	RBFOX1	0
T	C	0.015940980	5.568799794	3.531186463	MED24	0
T	C	0.043299593	4.039347955	3.441312279	KANSL1	0
A	G	0.000817182	-5.50787070	5.407641742	RPS3AP49	4825
A	G	0.001675887	-5.29319139	4.161763008	AC007773.2	0
T	C	0.047721764	-4.84159665	3.201740486	NCOA5	0
T	G	0.047539562	-3.99907696	3.704749189	ZFP64	0
A	G	0.044165133	-4.75545496	3.227905871	HORMAD2	0
T	C	0.046228798	4.010696075	3.677100627	DDX17	0

func	CADD	RDB	minChrState	PVAL_in_NE	BETA_in_NE	SE_in_NEUR
intronic	0.799	5	5	1.73e-06	0.0111	0.0023
ncRNA_exon	5.266	NA	5	4.324e-05	0.011	0.0027
intronic	3.884	7	4	4.366e-05	0.0105	0.0026
exonic	2.547	1f	3	3.075e-05	0.0095	0.0023
intronic	13.51	NA	1	1.873e-05	-0.0102	0.0024
UTR3	5.735	6	4	3.74e-05	0.0113	0.0027
intronic	0.479	NA	4	3.222e-12	-0.0161	0.0023
intronic	5.43	5	5	1.439e-05	0.0133	0.0031
exonic	0.558	NA	4	5.692e-08	-0.0135	0.0025
intronic	0.096	5	1	3.908e-05	0.012	0.0029
intronic	2.957	NA	4	1.265e-06	0.0124	0.0026
intergenic	4.386	5	5	2.155e-06	0.0107	0.0023
intronic	14.55	4	4	1.133e-07	0.0123	0.0023
intronic	4.626	5	5	3.998e-06	-0.0226	0.0049
intronic	7.011	4	5	9.817e-07	-0.0111	0.0023
intronic	2.236	4	5	5.511e-08	-0.0133	0.0024
intergenic	0.544	1f	5	7.581e-10	-0.0146	0.0024
intronic	1.141	1f	2	2.043e-13	-0.0173	0.0024
intronic	0.41	5	4	1.659e-05	-0.0107	0.0025
intronic	0.57	7	4	6.739e-12	0.0156	0.0023
ncRNA_intro	0.013	6	5	3.703e-05	-0.011	0.0027
intronic	4.822	6	1	2.565e-08	0.0131	0.0023
intronic	5.833	1d	4	5.36e-05	0.0118	0.0029
ncRNA_intro	3.068	6	7	3.632e-08	-0.0153	0.0028
ncRNA_intro	2.202	5	4	1.202e-07	-0.0161	0.003
intronic	4.733	1f	4	1.288e-06	-0.011	0.0023
intronic	1.046	7	5	6.359e-05	-0.0092	0.0023
intronic	3.438	7	7	1.98e-06	-0.0108	0.0023
intronic	4.035	2c	1	6.054e-05	0.0094	0.0024

PVAL_in_HD	BETA_in_HD	SE_in_HDL
0.0003238	0.0148	0.0034
0.0007401	0.0144	0.0042
0.001341	0.0189	0.0054
0.0005723	0.0142	0.0036
0.001192	0.0125	0.0039
0.0009901	0.0169	0.006
0.0002426	0.0137	0.0034
0.0001787	0.0266	0.0066
6.902e-06	0.0159	0.0037
0.0009294	0.0145	0.0044
3.125e-05	0.0251	0.0053
0.001053	0.0111	0.0034
5.707e-13	0.0263	0.0035
0.0008733	0.0257	0.0079
1.569e-05	0.0145	0.0034
7.015e-07	0.0189	0.0036
1.877e-22	0.0354	0.0036
6.031e-11	0.0237	0.0035
0.0001698	0.0249	0.0059
0.0009924	0.0095	0.0034
0.00127	0.0171	0.0056
0.0004137	0.0186	0.005
0.0005789	0.0174	0.0045
6.386e-08	0.0248	0.0043
3.158e-05	0.0309	0.0063
0.001366	0.0155	0.0048
0.0002116	0.0161	0.0049
0.001247	0.0164	0.0048
0.0002359	0.0136	0.0036

locusnum	CHR	LEAD_SNP	LEAD_BP	MinBP	MaxBP	Overlapping
1	3	rs2526392	50191191	50184538	50412945	SBP,DBP
2	3	rs4687625	52563718	52536514	52838402	CAD,BMI,LDL
3	3	rs9853387	136038988	135798730	136503896	CAD,BMI,TG
4	6	rs2072633	31919578	31288877	32739174	CAD,BMI,LDL
5	6	rs9377232	101240430	100953047	101426759	BMI
6	7	rs6948810	21523194	21474610	21555536	BMI,LDL
7	7	rs10239225	43658789	43618688	43881918	
8	11	rs4752801	47907641	47229316	47946836	BMI,LDL,T2D
9	11	rs866901	77926309	77909014	78135704	CAD,BMI,LDL
10	12	rs10850379	110002777	109794737	110052245	
11	12	rs982539	125821545	125807971	125822358	
12	14	rs12588415	75278211	75120628	75378185	HDL,SBP,DBP
13	16	rs1002252	71286676	71278016	71376751	LDL
14	17	rs12945939	8253984	8223226	8260870	
15	17	rs12309	38175462	38122708	38219005	LDL,HDL
16	17	rs1230065	43461481	43461460	43534322	
17	17	rs1980118	64520446	64442183	64520446	

A1	A2	FDR	Z_in_NEUR	Z_in_TC	nearestGene	dist
T	C	0.038283268	4.471040374	3.374946657	RP11-493K19	0
T	C	0.021554874	-4.79398561	3.55686983C	NT5DC2	0
A	G	0.008607397	-4.98578731	3.832398425	PCCB	0
A	G	0.000109862	-6.04180456	5.294106073	CFB:CFB	00:00
A	G	0.016352282	-5.25552849	3.642121868	ASCC3	0
T	C	0.003380685	5.170234838	4.684459252	SP4	0
T	G	0.035130652	-4.39711347	3.53922740C	STK17A:COA	00:00
A	G	0.00010502C	6.052404046	5.106777055	NUP160	37533
T	C	0.04335039C	-4.31225213	4.000046644	GAB2	33
T	C	3.36079414C	7.015151452	5.123321396	MMAB	0
A	G	0.021988339	4.966676683	3.551048296	TMEM132B	0
A	G	0.003289989	6.863083093	4.105580959	YLPM1	0
T	C	0.034233803	-5.20614886	3.411316501	HYDIN	22050
A	G	0.047800391	-4.98065323	3.301031694	ODF4	4619
T	C	0.007867969	-4.91571280	5.203756379	MED24	0
T	G	0.020167706	-6.04762128	3.57772336C	ARHGAP27	9793
A	C	0.047194439	4.277976412	3.651315987	PRKCA	0

func	CADD	RDB	minChrState	PVAL_in_NE	BETA_in_NE	SE_in_NEUR
ncRNA_intro	5.956	NA	5	7.784e-06	0.0163	0.0036
intronic	1.704	1f	3	1.635e-06	-0.0109	0.0023
intronic	2.279	6	4	6.171e-07	-0.0119	0.0024
UTR3	2.404	NA	4	1.524e-09	-0.0137	0.0023
intronic	0.173	6	4	1.476e-07	-0.0119	0.0023
intronic	1.791	6	4	2.338e-07	0.0131	0.0025
intronic	8.759	7	4	1.097e-05	-0.0122	0.0028
intergenic	6.7	1d	2	1.427e-09	0.0143	0.0024
downstream	2.574	NA	4	1.616e-05	-0.0132	0.0031
intronic	4.895	6	4	2.297e-12	0.016	0.0023
intronic	11.76	5	5	6.811e-07	0.0116	0.0023
intronic	0.57	7	4	6.739e-12	0.0156	0.0023
intergenic	0.818	6	5	1.928e-07	-0.0136	0.0026
intergenic	3.973	1f	5	6.337e-07	-0.0117	0.0024
UTR3	1.122	NA	2	8.846e-07	-0.0116	0.0024
intergenic	5.517	3a	5	1.47e-09	-0.0137	0.0023
intronic	5.112	NA	4	1.886e-05	0.0106	0.0025

PVAL_in_TC	BETA_in_TC	SE_in_TC
0.0007383	0.0327	0.0082
0.0003753	0.0138	0.0035
0.0001269	0.0156	0.0037
1.196e-07	0.0199	0.0036
0.0002704	0.0147	0.0036
2.807e-06	0.0278	0.0058
0.0004013	0.016	0.0047
3.277e-07	0.0197	0.0038
6.333e-05	0.0207	0.0047
3.002e-07	0.0192	0.0035
0.0003837	0.0184	0.0054
4.033e-05	0.0146	0.0035
0.0006465	0.0141	0.004
0.0009633	0.0188	0.0054
1.953e-07	0.0258	0.0047
0.0003466	0.0137	0.0036
0.0002609	0.0212	0.0057

locusnum	CHR	LEAD_SNP	LEAD_BP	MinBP	MaxBP	Overlapping
1	1	rs1460940	72814617	72628347	72959039	BMI,WHR,T2
2	1	rs754666	230250182	230230169	230261441	
3	2	rs13010035	29764460	29760883	29779019	
4	3	rs9831321	136142592	135625498	136638975	CAD,BMI,TC,[illegible]
5	5	rs6421926	104075130	103783801	104082179	
6	6	rs2856674	32659645	26408472	32963948	WHR,SBP,PP
7	6	rs7759381	130391887	130374461	130422142	BMI
8	8	rs920047	11087475	10118827	11178093	BMI
9	11	rs10832027	13357183	13354509	13370535	CAD,BMI,HD
10	11	rs7480140	46856335	46714279	47242761	BMI,LDL,HDL
11	12	rs11046486	22755536	22747967	22777844	
12	16	rs1046276	30914626	30801183	31155458	BMI,DBP
13	18	rs538656	57850422	57728947	57913434	CAD,BMI,HD
14	19	rs10409835	32888660	32830261	32994338	HDL
15	20	rs17092148	33435161	32505658	33988114	CAD,BMI
16	22	rs5757161	38990662	38869463	39152412	HDL,CRP

A1	A2	FDR	Z_in_NEUR	Z_in_TG	nearestGene	dist
A	G	0.026715469	4.354616623	3.848589279	RPL31P12	47104
T	C	0.005932830	-4.89104897	3.953892394	GALNT2	0
T	C	0.022909257	4.412253996	3.690515557	ALK	0
A	G	0.002846117	5.172162666	4.092851838	STAG1	0
T	C	0.038922765	4.208299227	4.157961587	RP11-6N13.1	0
A	G	0.000963284	5.476794374	5.151145663	MTCO3P1	14255
A	G	0.045704542	-4.14306149	3.875284948	L3MBTL3	0
A	G	0.000464465	5.681139866	5.271653695	LINC00529	17659
A	G	0.001110451	-5.43398246	4.846592564	ARNTL	0
A	G	0.005493544	-4.91735809	4.001621037	CKAP5	0
T	C	0.011145085	4.671986892	4.014903983	ETNK1	22472
T	C	0.000508414	-5.85675451	4.500355214	CTF1	0
T	G	0.003233006	-5.13443026	4.060727702	RNU4-17P	19481
A	G	0.040503501	-5.29319139	3.358876619	AC007773.2	0
T	G	0.000741617	5.551394332	4.873388847	GGT7	0
A	G	0.014048949	4.588894288	4.797572609	FAM227A	0

func	CADD	RDB	minChrState	PVAL_in_NE	BETA_in_NE	SE_in_NEUR
intergenic	3.262	NA	15	1.333e-05	0.0124	0.0029
intronic	2.207	4	1	1.003e-06	-0.0113	0.0023
intronic	8.371	6	5	1.023e-05	0.011	0.0025
intronic	2.944	6	5	2.314e-07	0.0124	0.0024
ncRNA_intro	4.977	6	9	2.573e-05	0.0102	0.0024
intergenic	11.01	NA	7	4.331e-08	0.0174	0.0032
intronic	1.51	6	4	3.427e-05	-0.0107	0.0026
intergenic	1.164	NA	6	1.338e-08	0.2548	0.0449
intronic	2.236	4	5	5.511e-08	-0.0133	0.0024
intronic	4.127	6	4	8.772e-07	-0.0132	0.0027
ncRNA_intro	13.21	6	5	2.983e-06	0.013	0.0028
UTR3	6.07	4	1	4.72e-09	-0.0139	0.0024
intergenic	3.849	NA	5	2.83e-07	-0.0137	0.0027
ncRNA_intro	2.202	5	4	1.202e-07	-0.0161	0.003
intronic	4.563	1d	4	2.834e-08	0.0166	0.003
intronic	3.722	5	4	4.456e-06	0.011	0.0024

PVAL_in_TG	BETA_in_TG	SE_in_TG
0.0001188	0.0167	0.0043
7.689e-05	0.0144	0.0035
0.0002238	0.0205	0.0052
4.261e-05	0.0206	0.0048
3.211e-05	0.0214	0.005
2.589e-07	0.0364	0.0074
0.0001065	0.0215	0.0053
1.352e-07	0.0193	0.0035
1.256e-06	0.0175	0.0035
6.291e-05	0.0172	0.0039
5.947e-05	0.0202	0.0056
6.784e-06	0.0236	0.005
4.892e-05	0.0204	0.0051
0.0007826	0.0202	0.0062
1.097e-06	0.0303	0.0065
1.606e-06	0.0184	0.0035

locusnum	CHR	LEAD_SNP	LEAD_BP	MinBP	MaxBP	Overlapping
1	1	rs2815748	72816147	72628347	72959039	BMI,TG,WHR
2	2	rs41382648	43532524	43449385	43850357	WHR
3	3	rs352161	52221849	52217088	52467263	CAD,BMI,LDL
4	4	rs2892785	118984212	118976318	119152498	DBP
5	6	rs56043606	20893310	20893310	20913634	
6	6	rs6457617	32663851	32629859	32678260	LDL,HDL
7	6	rs10948071	43280713	43260660	43397259	DBP,PP
8	6	rs6919397	126659043	126623947	127080700	CAD,WHR,CR
9	8	rs2897754	4948514	4948514	4958358	
10	8	rs17150816	9790737	9374120	10009087	
11	10	rs11258384	13482031	13479684	13558641	
12	11	rs2904129	47715574	47385350	48693639	BMI,TC,HDL,I
13	11	rs117512134	72549345	72527227	72708204	
14	18	rs476828	57852587	57728947	57913434	CAD,BMI,TG,
15	20	rs6124969	45816277	45760083	45848043	

A1	A2	FDR	Z_in_NEUR	Z_in_T2D	nearestGene	dist
A	G	0.027874887	4.29613725C	3.867404243	RPL31P12	48634
T	C	0.008073402	4.504625801	-4.83975391	THADA	0
A	C	0.030115306	-4.03716942	-3.94440008	ALDOAP1	5336
A	G	0.048904589	-4.35461662	-3.68355901	NDST3	0
T	C	0.035834804	-3.97133831	-4.73412695	CDKAL1	0
T	C	0.031855528	4.015181759	3.867404243	MTCO3P1	10049
T	C	0.02668500C	-4.08127148	-3.97290960	ZNF318	0
T	G	0.00027221C	5.495185468	-5.45856431	CENPW	2276
A	G	0.038986091	5.876314894	-3.75987246	SNORA70	37286
A	C	0.020123013	4.185770812	-3.98669522	LINC00599	27860
T	C	0.038986091	4.132524824	-3.75987246	BEND7	0
T	C	0.029794235	-4.23941803	-3.84612614	AGBL2	0
A	G	0.024918398	4.107768631	-4.37758784	ATG16L2:FCH	00:00
T	C	0.001378212	5.175829992	-4.69241403	RNU4-17P	21646
T	C	0.033627312	-4.21761491	3.808168264	EYA2	0

func	CADD	RDB	minChrState	PVAL_in_NE	BETA_in_NE	SE_in_NEUR
intergenic	2.386	NA	7	1.738e-05	0.0123	0.0029
intronic	8.499	NA	2	6.649e-06	0.0149	0.0033
intergenic	4.039	NA	5	5.41e-05	-0.0092	0.0023
intronic	1.407	7	4	1.333e-05	-0.0147	0.0034
intronic	16.47	5	5	7.147e-05	-0.0218	0.0055
intergenic	7.247	6	9	5.94e-05	0.0091	0.0023
intronic	1.805	2c	2	4.479e-05	-0.0095	0.0023
intergenic	7.183	5	5	3.903e-08	0.0125	0.0023
intergenic	5.824	7	9	4.195e-09	0.0138	0.0023
intergenic	0.351	5	5	2.842e-05	0.0225	0.0054
intronic	6.845	2b	1	3.588e-05	0.0101	0.0025
intronic	3.67	7	5	2.241e-05	-0.0205	0.0048
UTR3	19.96	4	4	3.995e-05	0.022	0.0054
intergenic	0.786	NA	5	2.269e-07	0.0138	0.0027
intronic	7.987	7	4	2.469e-05	-0.01	0.0024

PVAL_in_T2I	BETA_in_T2I	SE_in_T2D
0.00011	0.06	0.015
1.3e-06	-0.094	0.019
8,00E-05	-0.049	0.012
0.00023	-0.065	0.018
2.2e-06	-0.14	0.029
0.00011	0.057	0.015
7.1e-05	-0.049	0.012
4.8e-08	-0.068	0.013
0.00017	-0.048	0.013
6.7e-05	-0.12	0.03
0.00017	-0.048	0.013
0.00012	-0.097	0.025
1.2e-05	-0.15	0.033
2.7e-06	-0.067	0.014
0.00014	0.048	0.013

locusnum	CHR	LEAD_SNP	LEAD_BP	MinBP	MaxBP	Overlapping
1	3	rs13091025	52467324	52277445	53101580	CAD,BMI,TC,[illegible]
2	3	rs9853387	136038988	135798730	136503896	CAD,TG,TC,H[illegible]
3	6	rs592229	31930441	31288877	32739174	CAD,BMI,TC,[illegible]
4	7	rs6948810	21523194	21474610	21555536	BMI,TC
5	8	rs2737219	116635688	116563879	116645056	BMI,HDL,DB[illegible]
6	11	rs4567413	47232184	46798631	47242761	BMI,TC,TG,H[illegible]
7	11	rs866901	77926309	77909014	78135704	CAD,BMI,TC,[illegible]
8	16	rs1002252	71286676	71278016	71376751	TC
9	17	rs12309	38175462	38122708	38219005	HDL,TC
10	17	rs1230065	43461481	43461460	43534322	CAD

A1	A2	FDR	Z_in_NEUR	Z_in_LDL	nearestGene	dist
A	C	0.023779502	4.641732479	3.671851072	SEMA3G	0
A	G	0.016661121	-4.98578731	3.779410631	PCCB	0
T	G	0.002447345	5.280119016	5.266432973	SKIV2L	0
T	C	0.003634706	5.170234838	4.401308518	SP4	0
A	G	0.002247843	5.303987619	4.371669629	TRPS1	0
T	C	0.030726261	-4.89046117	3.590298095	DDB2	4308
T	C	0.046614184	-4.31225213	3.457852205	GAB2	33
T	C	0.015894070	-5.20614886	3.792563678	HYDIN	22050
T	C	0.008522000	-4.91571280	4.134841305	MED24	0
T	G	0.019594955	-6.04762128	3.730895974	ARHGAP27	9793

func	CADD	RDB	minChrState	PVAL_in_NE	BETA_in_NE	SE_in_NEUR
UTR3	15.3	4	2	3.455e-06	0.0163	0.0035
intronic	2.279	6	4	6.171e-07	-0.0119	0.0024
intronic	4.434	1f	4	1.291e-07	0.0121	0.0023
intronic	1.791	6	4	2.338e-07	0.0131	0.0025
intronic	14.55	4	4	1.133e-07	0.0123	0.0023
intergenic	2.629	5	5	1.006e-06	-0.0118	0.0024
downstream	2.574	NA	4	1.616e-05	-0.0132	0.0031
intergenic	0.818	6	5	1.928e-07	-0.0136	0.0026
UTR3	1.122	NA	2	8.846e-07	-0.0116	0.0024
intergenic	5.517	3a	5	1.47e-09	-0.0137	0.0023

PVAL_in_LDI	BETA_in_LDI	SE_in_LDL
0.0002408	0.0239	0.0058
0.0001572	0.0156	0.0038
1.391e-07	0.0212	0.004
1.076e-05	0.0269	0.0059
1.233e-05	0.0173	0.0038
0.0003303	0.0203	0.0057
0.0005445	0.0188	0.0048
0.0001491	0.0167	0.0042
3.552e-05	0.0207	0.005
0.0001908	0.0142	0.0037

locusnum	CHR	LEAD_SNP	LEAD_BP	MinBP	MaxBP	Overlapping
1	6	rs592229	31930441	31564167	32649676	CAD,BMI,LDL
2	6	rs6919397	126659043	126623947	127080700	CAD,WHR,T2
3	8	rs10102524	11534907	8088230	11830150	SBP,DBP,PP
4	11	rs1037169	13361005	13354509	13370535	CAD,TG
5	11	rs11601694	47614575	47385350	49128599	TC,LDL,HDL,T
6	11	rs198457	61471678	61448384	61525020	
7	15	rs3985865	48041486	48017489	48083383	
8	18	rs663640	57846077	57732418	57912226	CAD,BMI,HD
9	22	rs4821797	39016182	38869463	39152412	HDL,TG
10	22	rs9611550	41707054	41215672	42216326	BMI

A1	A2	FDR	Z_in_NEUR	Z_in_CRP	nearestGene	dist
T	G	0.000868445	5.280119016	4.733333333	SKIV2L	0
T	G	0.000362778	5.495185468	5.390243902	CENPW	2276
A	G	6.9754434411	5.863320966	-5.90476190	GATA4	0
T	C	0.000353413	5.501487648	-6.11363636	ARNTL	0
T	C	0.010077458	4.607010185	4.220338983	C1QTNF4	0
T	C	0.036909472	-4.24809081	3.785714285	DAGLA	0
T	C	0.024617828	-4.65607152	3.902439024	SEMA6D	0
T	C	0.015993610	-5.13041766	4.019230769	RNU4-17P	15136
T	C	0.005995052	-4.75916030	5.604651162	FAM227A	0
A	C	0.014516157	4.692414035	4.043478260	ZC3H7B	0

**func**	**CADD**	**RDB**	**minChrState**	**PVAL_in_NE**	**BETA_in_NE**	**SE_in_NEUR**
intronic	4.434	1f	4	1.291e-07	0.0121	0.0023
intergenic	7.183	5	5	3.903e-08	0.0125	0.0023
intronic	0.168	4	1	4.537e-09	0.2516	0.0429
intronic	0.535	NA	2	3.766e-08	0.0135	0.0025
intronic	5.472	2b	2	4.085e-06	0.0255	0.0055
intronic	1.025	NA	4	2.156e-05	-0.0125	0.0029
intronic	0.35	6	4	3.223e-06	-0.0106	0.0023
intergenic	4.344	NA	5	2.891e-07	-0.0141	0.0027
intronic	2.465	7	4	1.944e-06	-0.0114	0.0024
intronic	0.355	7	4	2.7e-06	0.2159	0.046

PVAL_in_CRI	BETA_in_CRI	SE_in_CRP
2.20862328{	0.0213	0.0045
7.03621212{	0.0221	0.0041
3.53156413{	-0.0248	0.0042
9.738607871	-0.0269	0.0044
2.43935236{	0.0498	0.0118
0.00015326	0.0212	0.0056
9.52282322{	0.016	0.0041
5.83884603{	0.0209	0.0052
2.08674804{	0.0241	0.0043
5.26640412{	0.0186	0.0046

locusnum	CHR	LEAD_SNP	LEAD_BP	MinBP	MaxBP	Overlapping [illegible]
1	1	rs6692693	8707116	8431607	8892577	BMI,WHR
2	2	rs736699	26913930	26911509	26932796	SBP,DBP,BMI
3	2	rs343968	44956340	44905806	45004016	SBP
4	2	rs848286	58394543	58007905	58674393	SBP,BMI
5	2	rs72932707	203639395	203639395	204196618	CAD,BMI
6	3	rs6788993	52605136	52277445	52838402	CAD,BMI,HDL
7	3	rs12637791	85525323	85403892	85784084	SBP,BMI
8	3	rs7641355	107035683	106986380	107072548	
9	4	rs16854051	42123892	41879969	42161491	SBP
10	5	rs4269288	122766938	122650049	122803786	BMI
11	6	rs2856674	32659645	25450026	32963948	WHR,TG
12	6	rs10948071	43280713	43260660	43397259	T2D
13	6	rs9399383	142021034	141864198	142033527	
14	7	rs17165701	12239274	12212919	12286050	SBP
15	8	rs2921372	8921727	8524474	10277982	DBP,CRP
16	8	rs3134112	110104855	110058677	110106809	
17	8	rs7813434	116511856	116464988	116632819	DBP,HDL,BMI
18	9	rs11791636	23819940	23805555	23827667	WHR
19	9	rs10821154	96302506	96155812	96381916	SBP
20	9	rs7851784	126981390	126978191	126985438	
21	9	rs4838254	128008537	127766897	128399285	SBP
22	10	rs11000925	75972601	75867193	76421529	SBP,CAD,BMI
23	10	rs77335224	104636276	104487871	105059896	DBP,CAD,BM[illegible]
24	11	rs3180446	45263340	45203212	45345244	SBP,BMI
25	11	rs7107356	47676170	47401448	49128599	DBP,BMI,CRP
26	11	rs79260745	57612960	57369008	57681828	
27	11	rs2450122	77930345	77909014	78135704	SBP,CAD,BMI
28	11	rs10765189	88874884	88768842	89057130	
29	12	rs79601649	49762365	49737114	50160662	SBP
30	14	rs1866628	75075505	75057809	75113506	SBP
31	15	rs4886937	78139187	78076272	78152626	BMI
32	15	rs17514846	91416550	91412850	91429042	DBP,CAD
33	16	rs11642627	6400688	6388400	6412487	
34	16	rs923154	77055434	77050826	77195686	
35	17	rs2165846	44941366	44941366	44947821	SBP
36	18	rs4522497	42095022	41978081	42142620	

A1	A2	FDR	Z_in_NEUR	Z_in_PP	nearestGene	dist
T	G	0.018492700	4.282506177	-4.14199356	RERE	0
A	G	0.023402320	4.189637856	7.423111950	KCNK3	1688
A	G	0.047924140	-3.89234482	3.615559176	CAMKMT	0
T	C	0.026968768	5.452308867	-3.82645860	FANCL	0
T	C	0.026815801	4.135164800	-5.48294630	ICA1L	1294
T	C	0.040067990	4.297416912	-3.68069401	SMIM4:PBRM	00:00
T	G	0.006498643	-5.00576709	-4.28971816	CADM2	0
T	C	0.016895063	-4.43226109	3.987333736	LINC00883	0
T	C	0.032259391	-5.43567296	3.761792471	BEND4	0
A	C	0.045853849	-4.89325849	3.629200832	CEP120	7651
A	G	0.000385193	5.476794374	-7.34201614	MTCO3P1	14255
T	C	0.030612782	-4.08127148	-5.54827612	ZNF318	0
A	G	0.049765745	3.876201109	-3.67451676	RPS3AP23	63393
T	C	0.003022036	-6.05105534	-4.50904531	TMEM106B	11592
A	G	0.012429604	-4.81731896	4.088324541	ERI1	0
A	G	0.028150020	4.114809086	4.390866200	TRHR	0
A	C	0.024224175	4.175142180	-3.92809890	TRPS1	0
T	C	0.032965751	-4.68062619	-3.75376239	ELAVL2	0
A	G	0.006344042	5.231434098	-4.29703227	FAM120A	0
A	C	0.014388213	4.442474924	-4.04013694	NEK6	38494
A	G	0.002469949	-4.98786564	4.575448877	HSPA5	4927
A	G	0.028351121	-4.11173316	4.493566912	ADK	0
T	C	0.001007894	-5.23859919	-10.1011645	C10orf32-ASM	00:00
T	C	0.014837276	-4.74001727	4.030492943	SYT13	0
A	G	0.004794990	-7.04747006	-4.37813354	AGBL2	4972
T	C	0.008082961	-6.35590726	4.223441352	OR5BA1P	20809
T	C	0.018935652	4.273532760	4.053903506	GAB2	0
T	C	0.043736564	3.931863283	-3.86673980	TYR	35735
A	G	0.007319787	4.760201265	-4.25398433	SPATS2	0
T	C	0.007120124	4.641374024	4.316531599	LTBP2	0
T	C	0.046607135	5.669997024	3.622738924	LINGO1	25944
A	C	0.013621433	-4.43830518	4.059063098	FURIN	0
T	C	0.029217297	4.099636250	-4.03943549	RBFOX1	0
T	C	0.019065896	4.366938823	-3.94717123	MIR4719	152517
A	G	0.004863485	4.773223944	5.470250476	WNT9B	0
T	C	0.049870055	3.875284948	3.871652481	CTC-782O7.1	0
			23			
			13			

**func**	**CADD**	**RDB**	**minChrState**	**posMapFilt**	**eqtlMapFilt**	**ciMapFilt**
intronic	2.817	5	4	1	0	1
intergenic	0.475	5	5	1	0	0
intronic	3.298	7	5	1	0	0
intronic	1.87	NA	4	1	0	0
upstream	4.065	5	4	1	1	0
intronic	2.813	7	4	1	1	0
intronic	11.94	6	5	1	0	0
ncRNA_intror	3.015	7	5	0	0	0
intronic	0.06	7	4	1	1	0
intergenic	9.069	7	5	1	0	0
intergenic	11.01	NA	7	0	1	0
intronic	1.805	2c	2	1	0	0
intergenic	3.191	6	9	0	0	0
intergenic	10.51	4	2	0	1	0
intergenic	2.019	NA	5	1	1	0
intronic	3.241	6	9	1	0	0
intronic	0.459	5	5	1	1	0
intronic	4.062	5	1	1	0	0
intronic	0.376	7	4	1	0	0
intergenic	2.937	1f	1	0	0	0
ncRNA_intror	0.115	6	5	1	1	0
intronic	0.692	7	5	1	0	0
intronic	3.704	7	4	1	1	0
UTR3	2.071	NA	5	1	1	0
intergenic	3.079	6	13	1	1	0
intergenic	9.605	6	5	0	1	0
exonic	12.65	NA	4	1	1	0
intergenic	6.025	7	5	0	0	0
intronic	13.83	4	1	1	0	1
intronic	3.446	4	2	1	1	0
intergenic	0.908	6	5	0	0	0
intronic	10.53	4	1	1	1	0
ncRNA_intror	2.722	5	5	1	0	0
intergenic	0.646	7	5	0	1	0
intronic	12.21	1f	5	1	1	0
ncRNA_intror	1.978	5	5	0	0	0

PVAL_in_NEU	BETA_in_NEU	SE_in_NEUR	PVAL_in_PP	BETA_in_PP
1.848e-05	0.0101	0.0024	3.443e-05	-0.1204
2.794e-05	0.0098	0.0023	1.144e-13	0.2258
9.928e-05	-0.0118	0.003	0.0002997	0.147
4.972e-08	0.0128	0.0024	0.00013	-0.1183
3.547e-05	0.014	0.0034	4.183e-08	-0.2678
1.728e-05	0.0099	0.0023	0.0002326	-0.1045
5.564e-07	-0.0118	0.0024	1.789e-05	-0.1261
9.325e-06	-0.0111	0.0025	6.682e-05	0.1212
5.459e-08	-0.0155	0.0028	0.0001687	0.1356
9.918e-07	-0.0132	0.0027	0.0002843	0.1236
4.331e-08	0.0174	0.0032	2.104e-13	-0.3296
4.479e-05	-0.0095	0.0023	2.885e-08	-0.1731
0.0001061	0.0088	0.0023	0.0002383	-0.1055
1.439e-09	-0.0151	0.0025	6.512e-06	-0.1547
1.455e-06	-0.2079	0.0431	4.345e-05	0.1251
3.875e-05	0.0095	0.0023	1.129e-05	0.1276
2.978e-05	0.0095	0.0023	8.562e-05	-0.1115
2.86e-06	-0.0148	0.0032	0.0001742	-0.2768
1.682e-07	0.0125	0.0024	1.731e-05	-0.1381
8.893e-06	0.011	0.0025	5.342e-05	-0.1238
6.105e-07	-0.0115	0.0023	4.752e-06	0.1507
3.927e-05	-0.0138	0.0034	7.004e-06	0.1668
1.618e-07	-0.0212	0.004	5.459e-24	-0.4684
2.137e-06	-0.0113	0.0024	5.566e-05	0.1259
1.822e-12	-0.0159	0.0023	1.197e-05	-0.1236
2.072e-10	-0.0153	0.0024	2.406e-05	0.136
1.924e-05	0.0133	0.0031	5.037e-05	0.1481
8.429e-05	0.0095	0.0024	0.0001103	-0.1204
1.934e-06	0.0199	0.0042	2.1e-05	-0.213
3.461e-06	0.0106	0.0023	1.585e-05	0.1237
1.428e-08	0.0143	0.0025	0.0002915	0.1194
9.067e-06	-0.0102	0.0023	4.927e-05	0.1234
4.138e-05	0.0134	0.0033	5.358e-05	-0.1832
1.26e-05	0.0105	0.0024	7.908e-05	-0.1269
1.813e-06	0.0114	0.0024	4.494e-08	0.1663
0.0001065	0.0103	0.0026	0.0001081	0.1319

locusnum	CHR	LEAD_SNP	LEAD_BP	MinBP	MaxBP	Overlapping
1	1	rs10464	201792306	201760610	201887721	CAD, BMI

A1	A2	FDR	Z_in_NEUR	Z_in_CIGPER	nearestGene	dist
T	C	0.000761925	5.395665580	4.771675155	NAV1:IPO9-A	00:00

func	CADD	RDB	minChrState	PVAL_in_NE	BETA_in_NE	SE_in_NEUR
ncRNA_intro	10.55	5	4	6.827e-08	0.0138	0.0026

PVAL_in_CIG	BETA_in_CIG	SE_in_CIGPERDAY
1.827e-06	0.4344	0.091

Phenotype	CHR	LEAD_SNP	LEAD_BP	MinBP	MaxBP	A1	A2
BMI, TG,WHR,SBP AND	1	rs1460940	72814617	72628347	72959392	A	G
SBP,DBP,BMI AND PP	2	rs736699	26913930	26911509	26932796	A	G
SBP AND PP	2	rs343968	44956340	44905806	45004016	A	G
SBP,BMI AND PP	2	rs848286	58394543	58007905	58674393	T	C
CAD,BMI AND PP	2	rs72932707	203639395	203639395	204196618	T	C
HDL AND DBP	2	rs6738482	61494901	61242410	61837947	A	G
WHR AND DBP	2	rs17741344	148557573	148457576	148853296	T	C
TC AND LDL	3	rs9853387	136038988	135798730	136503896	A	G
CAD,BMI,HDL,LDL,TC AI	3	rs6788993	52605136	52277445	52838402	T	C
SBP,BMI AND PP	3	rs12637791	85525323	85403892	85784084	T	G
TC,DBP AND SBP	3	rs1989839	50378946	50184538	50420554	A	G
CAD, DBP AND SBP	4	rs4691707	156441314	156420605	156443314	A	G
SBP AND PP	4	rs16854051	42123892	41879969	42161491	T	C
BMI, DBP AND SBP	4	rs11722027	144050017	144028173	144215346	A	G
CAD,T2D AND DBP	4	rs17516389	119239267	118976252	119264162	T	C
BMI AND PP	5	rs4269288	122766938	122650049	122803786	A	C
T2D, DBP,SBP AND PP	6	rs10948071	43280713	43260660	43397259	T	C
WHR,TG, LDL,CRP AND	6	rs2856674	32659645	25450026	32963948	A	G
HDL,CRP,LDL, TC,TG AN	6	rs2269426	32076499	31578772	32189481	A	G
CAD,WHR,CRP AND SBP	6	rs1490384	126851160	126659043	127080700	T	C
CAD,HDL,LDL,WHR,BMI	6	rs1077393	31610529	30997692	32189481	A	G
T2D AND DBP	6	rs2396004	43355851	43262303	43364494	A	G
CAD,WHR,CRP,T2D ANC	6	rs6925689	126865884	126623947	127080700	T	C
TC AND LDL	7	rs6948810	21523194	21474610	21555536	T	C
SBP AND PP	7	rs17165701	12239274	12212919	12286050	T	C
CAD AND SBP	7	rs58673065	1885600	1843200	2110850	A	G
CAD AND SBP	7	rs6460902	12255511	12200060	12285140	A	G
DBP,HDL,BMI,LDL AND	8	rs7813434	116511856	116464988	116632819	A	C
CRP, DBP, SBP AND PP	8	rs2736313	11086942	8088230	12199830	T	C
WHR AND PP	9	rs11791636	23819940	23805555	23827667	T	C
SBP AND PP	9	rs10821154	96302506	96155812	96381916	A	G
SBP AND PP	9	rs4838254	128008537	127766897	128399285	A	G
SBP,CAD,BMI AND PP	10	rs11000925	75972601	75867193	76421529	A	G
T2D, DBP AND SBP	10	rs10906382	13516426	13479684	13611368	T	C
CAD, BMI,DBP, SBP ANC	10	rs77335224	104636276	104487871	105059896	T	C
BMI, LDL AND TC	11	rs866901	77926309	77909014	78135704	T	C
HDL OG TG	11	rs10832027	13357183	13354509	13370535	A	G
SBP,BMI AND PP	11	rs3180446	45263340	45203212	45345244	T	C
SBP,CAD,BMI,LDL,TC AI	11	rs2450122	77930345	77909014	78135704	T	C
HDL AND SBP	11	rs1988724	10239723	9958403	10370675	A	C
BMI AND SBP	11	rs11038371	45282531	45258966	45345244	A	G
BMI,T2D,CRP,TC, TG, HI	11	rs7107356	47676170	47175327	49128599	A	G
CAD,BMI,LDL,TC AND SI	11	rs990706	78135704	77909014	78271614	T	C
SBP AND PP	12	rs79601649	49762365	49737114	50160662	A	G
HDL OG TC	14	rs12588415	75278211	75120628	75378185	A	G
TC, SBP AND PP	14	rs1866628	75075505	75057809	75113506	T	C
HDL,TC AND DBP	14	rs8004084	75220107	75144618	75377692	T	C
BMI AND PP	15	rs4886937	78139187	78076272	78152626	T	C

CAD, SBP, DBP AND PP	15	rs17514846	91416550	91412850	91429042	A	C
BMI AND SBP	15	rs7176782	69477380	69415482	69569464	A	G
CAD AND SBP	15	rs17514846	91416550	91412850	91429042	A	C
TC AND LDL	16	rs1002252	71286676	71278016	71376751	T	C
BMI,TG AND DBP	16	rs1549299	31154146	30916129	31155458	A	G
TC AND LDL	17	rs12309	38175462	38122708	38219005	T	C
TC AND LDL	17	rs1230065	43461481	43461460	43534322	T	G
SBP AND PP	17	rs2165846	44941366	44941366	44947821	A	G
CAD AND DBP	17	rs55938136	43798360	43798360	43798360	A	G
CAD,BMI,WHR,HDL	18	rs17700144	57811982	57728947	57987859	A	G
HDL OG TG	19	rs10409835	32888660	32830261	32994338	A	G
BMI AND WHR	19	rs9636202	18449238	18449238	18474892	A	G

FDR	Z_in_NEUR	Z_in_BMI	nearestGene	dist	func	CADD
0.0113909013963	4.35461662317	-9.37536015357	RPL31P12	47104	intergenic	3.262
0.023402320816020¢	4.18963785615	7.42311195043	KCNK3	1688	intergenic	0.475
0.047924140061262З	-3.89234482431	3.61555917689	CAMKMT	0	intronic	3.298
0.026968768823152	5.45230886725	-3.82645860208	FANCL	0	intronic	1.87
0.026815801232087	4.13516480072	-5.48294630951	ICA1L	1294	upstream	4.065
0.006263684508966	4.45360597614	-4.2698551013	USP34	0	intronic	2.003
0.010959481628421	5.54325499552	4.08096038229	ACVR2A	44512	intergenic	3.268
0.00860739735886	-4.98578731358	3.83239842588	PCCB	0	intronic	2.279
0.040067990192099	4.29741691262	-3.6806940116	SMIM4:PBRM1	00:00	intronic	2.813
0.006498643634386	-5.00576709809	-4.28971816401	CADM2	0	intronic	11.94
0.040461981747828	-5.10311943356	-3.55799248676	ZMYND10-AS1:ZI	00:00	ncRNA_intronic	11.3
0.0448918004809	-4.35330561706		MTND1P22	56338	intergenic	0.347
0.032259391339571	-5.43567296604	3.76179247161	BEND4	0	intronic	0.06
0.028960675808850	3.94478982621	-5.83441381676	RP11-284M14.1	0	ncRNA_intronic	2.192
0.026070249744127	-4.33016153627	3.75247113278	PRSS12	0	intronic	8.097
0.045853849627911	-4.89325849576	3.62920083278	CEP120	7651	intergenic	9.069
0.0266850005986	-4.08127148845	-3.97290960086	ZNF318	0	intronic	1.805
0.000385193550874	5.47679437498	-7.3420161496	MTCO3P1	14255	intergenic	11.01
0.000103104080835	7.85464688518	5.29579456786	TNXB:ATF6B	00:00	intronic	0.475
0.001090772280067	-5.2537909447	4.72386856607	MIR588	45300	intergenic	18.6
7.52269613861332e-	-5.66008277462	-5.36428610526	BAG6	0	intronic	5.552
0.041562395920894	3.72892286408	4.12248931577	ZNF318	18634	intergenic	2.553
0.002866985571603	4.68322226982	-4.50713121874	RNU6-200P	45548	intergenic	2.754
0.0033806858455	5.17023483889	4.68445925232	SP4	0	intronic	1.791
0.003022036127711	-6.05105534044	-4.50904531426	TMEM106B	11592	intergenic	10.51
0.007608568064580	4.4437848434	4.28383398987	MAD1L1	0	intronic	4.431
0.028238536654762	5.92306285077	3.70415087386	TMEM106B	0	intronic	0.71
0.024224175879593	4.17514218028	-3.92809890718	TRPS1	0	intronic	0.459
7.40004965922311e-	-5.84975419193	5.49518546859	LINC00529	18192	intergenic	1.004
0.032965751523945	-4.68062619372	-3.75376239133	ELAVL2	0	intronic	4.062
0.006344042042381	5.23143409848	-4.2970322745	FAM120A	0	intronic	0.376
0.002469949695261	-4.98786564489	4.57544887739	HSPA5	4927	ncRNA_intronic	0.115
0.028351121902289	-4.11173316994	4.49356691272	ADK	0	intronic	0.692
0.009473172738990	-4.36400954759	4.35232724956	BEND7	0	intronic	3.595
0.000739734238549	-5.23859919803	-11.6737003952	C10orf32-ASMT:	00:00	intronic	3.704
0.0176178768831	-4.31225213337	-3.2649070583	GAB2	33	downstream	2.574
0.00105275574367	-5.43398246143	4.96094791988	ARNTL	0	intronic	2.236
0.014837276912674	-4.74001727338	4.03049294369	SYT13	0	UTR3	2.071
0.018935652424117	4.27353276097	4.05390350633	GAB2	0	exonic	12.65
0.004048737644866	4.6699316277	4.81567925849	SBF2	0	intronic	3.537
0.023673621896534	4.02407371564	-4.129849996	SYT13	0	intronic	0.436
3.0776391269034e-0	-7.04747006968	-6.46799771416	AGBL2	4972	intergenic	3.079
0.022604796964901	-4.17713795565	-3.7912350567	RP11-452H21.4	0	ncRNA_exonic	1.788
0.007319787076423	4.76020126525	-4.25398433374	SPATS2	0	intronic	13.83
0.0360273563673	6.86308309352	3.29267249716	YLPM1	0	intronic	0.57
0.007120124902392	4.64137402432	4.31653159938	LTBP2	0	intronic	3.446
0.008002775461359	-6.54353852104	-4.18980035021	YLPM1	9961	intergenic	2.999
0.046607135734089	5.66999702405	3.62273892405	LINGO1	25944	intergenic	0.908

0.0136214336371354	-4.43830518151	4.0590630983	FURIN	0	intronic	10.53
0.0327003242782206	-3.94284707938	-3.64595557462	GLCE	0	intronic	0.391
0.0077230665111874	-4.43830518151	7.84020820402	FURIN	0	intronic	10.53
0.0342338039567	-5.2061488614	3.41131650178	HYDIN	22050	intergenic	0.818
0.0461991091082217	4.47612498022	-3.5188020677	PRSS36	0	exonic	14.24
0.00786796929331	-4.91571280463	5.2037563797	MED24	0	UTR3	1.122
0.0201677061463	-6.04762128386	3.57772336096	ARHGAP27	9793	intergenic	5.517
0.0048634856468952	4.77322394436	5.47025047698	WNT9B	0	intronic	12.21
0.0090754460653970	-11.5419461181	4.14676897218	CRHR1:RP11-105	00:00	ncRNA_intronic	3.353
0.000323560201216	-5.50787070534	14.6167615873	RPS3AP49	4825	ncRNA_intronic	3.068
0.0405035017783	-5.29319139928	3.35887661932	AC007773.2	0	ncRNA_intronic	2.202
0.033048758483	-3.89258923647	5.30658187615	PGPEP1	2158	intergenic	0.397

RDB	minChrState	PVAL_in_NEUR	BETA_in_NEUR	SE_in_NEUR	PVAL_in_BMI	BETA_in_BMI
NA	15	1.333e-05	0.0124	0.0029	6.894e-21	-0.0376
5	5	1	0	0 2.794e-05	0.0098	
7	5	1	0	0 9.928e-05	-0.0118	
NA	4	1	0	0 4.972e-08	0.0128	
5	4	1	1	0 3.547e-05	0.014	
6	4	1	1	0 8.444e-06	0.0119	
7	5	0	1	0 2.969e-08	0.0157	
6	4	6.171e-07	-0.0119	0.0024	0.0001269	0.0156
7	4	1	1	0 1.728e-05	0.0099	
6	5	1	0	0 5.564e-07	-0.0118	
NA	1	1	1	1 3.341e-07	-0.0182	
7	9	1.341e-05	-0.0104	0.0024	0.000554	
7	4	1	1	0 5.459e-08	-0.0155	
7	14	0	1	0 7.987e-05	0.0091	
7	4	1	0	0 1.49e-05	-0.0168	
7	5	1	0	0 9.918e-07	-0.0132	
2c	2	4.479e-05	-0.0095	0.0023	7.1e-05	-0.049
NA	7	0	1	0 4.331e-08	0.0174	
NA	1	1	1	0 4.009e-15	0.0185	
7	5	0	0	0 1.49e-07	-0.0119	
5	4	1	1	0 1.513e-08	-0.0128	
6	5	0	0	0 0.0001923	0.0085	
7	5	0	0	0 2.824e-06	0.0106	
6	4	2.338e-07	0.0131	0.0025	2.807e-06	0.0278
4	2	0	1	0 1.439e-09	-0.0151	
5	2	1	0	0 8.839e-06	0.012	
6	4	1	0	0 3.16e-09	0.0136	
5	5	1	1	0 2.978e-05	0.0095	
1f	5	0	1	0 4.923e-09	-0.2579	
5	1	1	0	0 2.86e-06	-0.0148	
7	4	1	0	0 1.682e-07	0.0125	
6	5	1	1	0 6.105e-07	-0.0115	
7	5	1	0	0 3.927e-05	-0.0138	
7	4	1	0	0 1.277e-05	-0.0108	
7	4	1	1	0 1.618e-07	-0.0212	
NA	4	1.616e-05	-0.0132	0.0031	0.001095	-0.0136
4	5	5.511e-08	-0.0133	0.0024	7.015e-07	0.0189
NA	5	1	1	0 2.137e-06	-0.0113	
NA	4	1	1	0 1.924e-05	0.0133	
NA	5	1	1	0 3.013e-06	0.0106	
6	5	1	1	0 5.72e-05	0.0092	
6	13	1	1	0 1.822e-12	-0.0159	
2b	2	1	1	0 2.952e-05	-0.0127	
4	1	1	0	1 1.934e-06	0.0199	
7	4	6.739e-12	0.0156	0.0023	0.0009924	0.0095
4	2	1	1	0 3.461e-06	0.0106	
7	7	1	1	0 6.008e-11	-0.0157	
6	5	0	0	0 1.428e-08	0.0143	

4	1	1	1	0	9.067e-06	-0.0102
5	5	1	1	0	8.052e-05	-0.0093
4	1	1	1	0	9.067e-06	-0.0102
6	5	1.928e-07	-0.0136	0.0026	0.0006465	0.0141
NA	1	1	1	0	7.601e-06	0.0111
NA	2	8.846e-07	-0.0116	0.0024	1.953e-07	0.0258
3a	5	1.47e-09	-0.0137	0.0023	0.0003466	0.0137
1f	5	1	1	0	1.813e-06	0.0114
6	5	1	1	0	8.107e-31	-0.0312
6	7	3.632e-08	-0.0153	0.0028	2.196e-48	0.0552
5	4	1.202e-07	-0.0161	0.003	0.0007826	0.0202
6	2	9.918e-05	-0.01	0.0026	1.117e-07	0.0191

SE_in_BMI		
0.004		
0.0023	1.144e-13	0.2258
0.003	0.0002997	0.147
0.0024	0.00013	-0.1183
0.0034	4.183e-08	-0.2678
0.0027	1.956e-05	-0.1346
0.0028	4.485e-05	0.1526
0.0037		
0.0023	0.0002326	-0.1045
0.0024	1.789e-05	-0.1261
0.0036	0.0003737	-0.1946
0.0028	0.0001687	0.1356
0.0023	5.398e-09	-0.218
0.0039	0.0001751	0.2004
0.0027	0.0002843	0.1236
0.012		
0.0032	2.104e-13	-0.3296
0.0024	1.185e-07	0.2003
0.0023	2.314e-06	0.1845
0.0023	8.127e-08	-0.1462
0.0023	3.748e-05	0.1252
0.0023	6.571e-06	-0.1341
0.0058		
0.0025	6.512e-06	-0.1547
0.0027	1.837e-05	0.1792
0.0023	0.0002121	0.1392
0.0023	8.562e-05	-0.1115
0.0441	3.903e-08	0.2324
0.0032	0.0001742	-0.2768
0.0024	1.731e-05	-0.1381
0.0023	4.752e-06	0.1507
0.0034	7.004e-06	0.1668
0.0025	1.347e-05	0.1856
0.004	1.737e-31	-0.7031
0.0042		
0.0036		
0.0024	5.566e-05	0.1259
0.0031	5.037e-05	0.1481
0.0023	1.467e-06	0.1856
0.0023	3.63e-05	-0.1541
0.0023	9.931e-11	-0.237
0.003	0.0001499	-0.1754
0.0042	2.1e-05	-0.213
0.0034		
0.0023	1.585e-05	0.1237
0.0024	2.792e-05	-0.1257
0.0025	0.0002915	0.1194

0.0023	4.927e-05	0.1234
0.0024	0.0002664	-0.1599
0.0023	4.498e-15	0.3087
0.004		
0.0025	0.0004335	-0.1168
0.0047		
0.0036		
0.0024	4.494e-08	0.1663
0.0027	3.372e-05	0.4091
0.0038		
0.0062		
0.0036		
