# Supplementary material for: Shared genetic architecture between neuroticism, coronary artery disease and cardiovascular risk factors
Source: Transl Psychiatry. 2021 Jun 17;11:368. doi: 10.1038/s41398-021-01466-9 (PMC8257646; doi:10.1038/s41398-021-01466-9)
Supplement: Supplementary file 2 — Supplementary Figure 1-32 [file 41398_2021_1466_MOESM2_ESM.pdf]

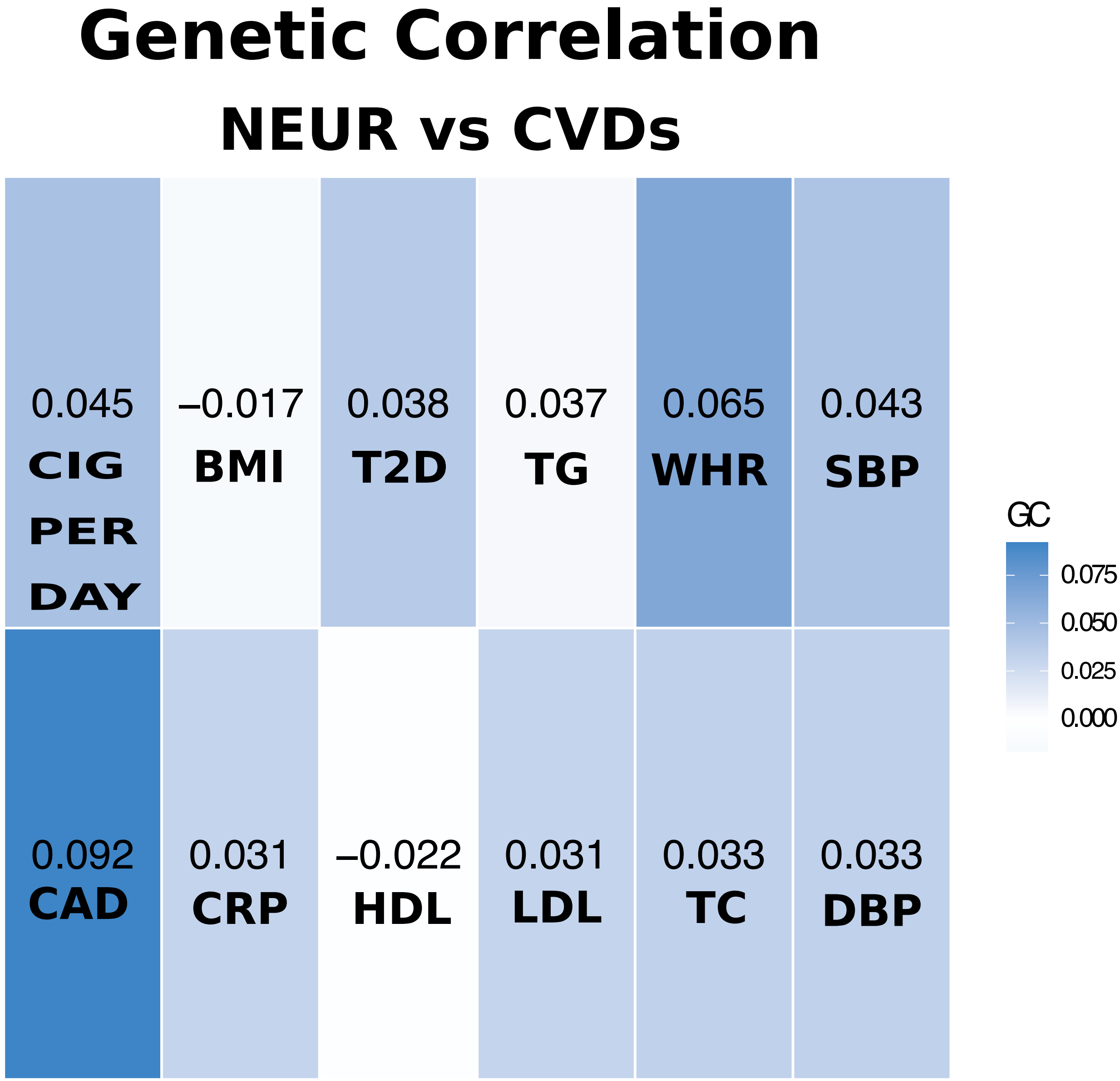
Genetic Correlation
NEUR vs CVDs
0.045
CIG PER DAY
−0.017
BMI
0.038
T2D
0.037
TG
0.065
WHR
0.043
SBP
0.092
CAD
0.031
CRP
−0.022
HDL
0.031
LDL
0.033
TC
0.033
DBP
GC
0.075
0.050
0.025
0.000

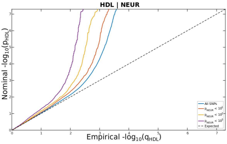

HDL | NEUR
Nominal -log10(pHDL)
Empirical -log10(qHDL)
All SNPs
pNEUR < 10^-1
pNEUR < 10^-2
pNEUR < 10^-3
Expected

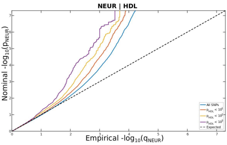

NEUR | HDL
Nominal -log10(pNEUR)
Empirical -log10(qNEUR)
All SNPs
PHDL < 10-1
PHDL < 10-2
PHDL < 10-3
Expected
0
1
2
3
4
5
6
7

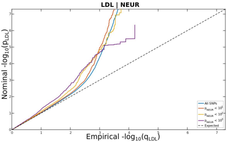

LDL | NEUR
Nominal -log10(pLDL)
Empirical -log10(qLDL)
All SNPs
pNEUR < 10^1
pNEUR < 10^2
pNEUR < 10^3
Expected

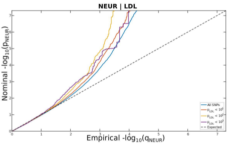
NEUR | LDL
Nominal -log10(pNEUR)
Empirical -log10(qNEUR)
All SNPs
PLDL < 10^1
PLDL < 10^2
PLDL < 10^3
Expected
0
1
2
3
4
5
6
7

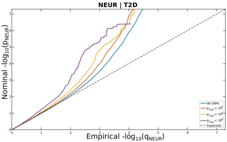

NEUR | T2D
Nominal -log10(pNEUR)
Empirical -log10(qNEUR)
All SNPs
PT2D < 10^1
PT2D < 10^2
PT2D < 10^3
Expected
0
1
2
3
4
5
6
7

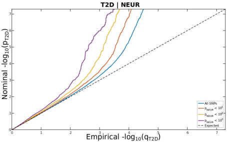

T2D | NEUR
Nominal -log10(pT2D)
Empirical -log10(qT2D)
All SNPs
pNEUR < 10^-1
pNEUR < 10^-2
pNEUR < 10^-3
Expected

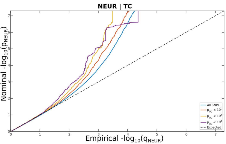
NEUR | TC
Nominal -log10(pNEUR)
Empirical -log10(qNEUR)
All SNPs
pTC < 10^-1
pTC < 10^-2
pTC < 10^-3
Expected

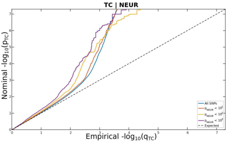
TC | NEUR
Nominal -log10(pTC)
Empirical -log10(qTC)
All SNPs
PNEUR < 10^1
PNEUR < 10^2
PNEUR < 10^3
Expected
0
1
2
3
4
5
6
7

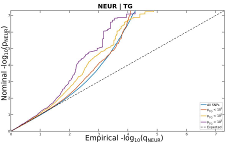

NEUR | TG
Nominal -log10(pNEUR)
Empirical -log10(qNEUR)
All SNPs
pTG < 10^-1
pTG < 10^-2
pTG < 10^-3
Expected

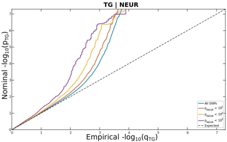
TG | NEUR
Nominal -log10(pTG)
Empirical -log10(qTG)
All SNPs
PNEUR < 10^1
PNEUR < 10^2
PNEUR < 10^3
Expected
0
1
2
3
4
5
6
7

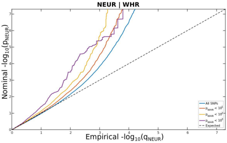

NEUR | WHR
Nominal -log10(pNEUR)
Empirical -log10(qNEUR)
All SNPs
pWHR < 10^1
pWHR < 10^2
pWHR < 10^3
Expected
0
1
2
3
4
5
6
7

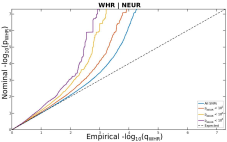

WHR | NEUR
Nominal -log10(pWHR)
Empirical -log10(qWHR)
All SNPs
PNEUR < 10^1
PNEUR < 10^2
PNEUR < 10^3
Expected

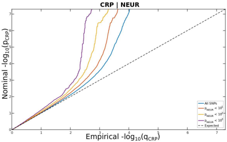
CRP | NEUR
Nominal -log10(pCRP)
Empirical -log10(qCRP)
All SNPs
PNEUR < 10^1
PNEUR < 10^2
PNEUR < 10^3
Expected
0
1
2
3
4
5
6
7

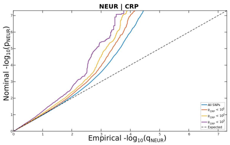
NEUR | CRP
Nominal -log10(pNEUR)
Empirical -log10(qNEUR)
All SNPs
pCRP < 10^-1
pCRP < 10^-2
pCRP < 10^-3
Expected

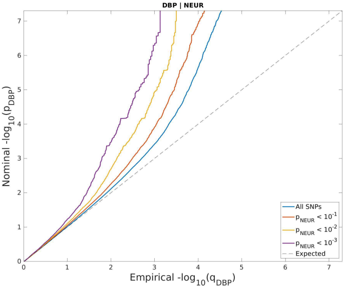

DBP | NEUR
Nominal -log10(pDBP)
Empirical -log10(qDBP)
All SNPs
pNEUR < 10-1
pNEUR < 10-2
pNEUR < 10-3
Expected
0
1
2
3
4
5
6
7

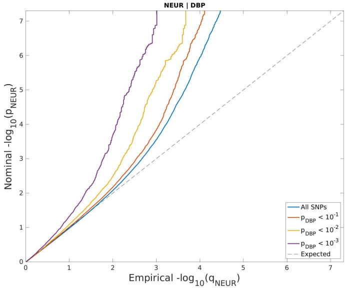

NEUR | DBP
Nominal -log10(pNEUR)
Empirical -log10(qNEUR)
All SNPs
pDBP < 10-1
pDBP < 10-2
pDBP < 10-3
Expected
0
1
2
3
4
5
6
7

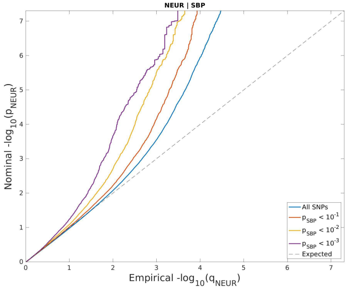

NEUR | SBP
Nominal -log$_{10}$(p$_{NEUR}$)
Empirical -log$_{10}$(q$_{NEUR}$)
All SNPs
$p_{SBP} < 10^{-1}$
$p_{SBP} < 10^{-2}$
$p_{SBP} < 10^{-3}$
Expected
0
1
2
3
4
5
6
7

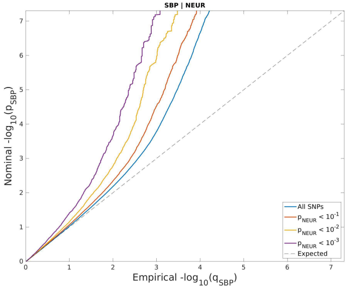

SBP | NEUR
Nominal -log10(pSBP)
Empirical -log10(qSBP)
All SNPs
pNEUR < 10-1
pNEUR < 10-2
pNEUR < 10-3
Expected

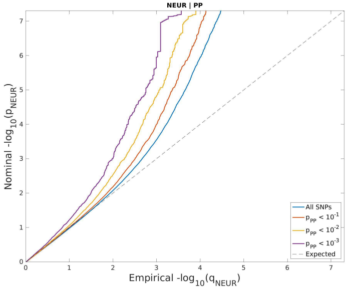

NEUR | PP
Nominal -log10(pNEUR)
Empirical -log10(qNEUR)
All SNPs
pPP < 10-1
pPP < 10-2
pPP < 10-3
Expected
0
1
2
3
4
5
6
7

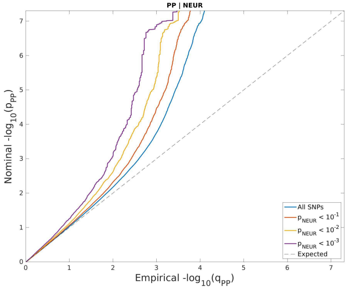
PP | NEUR
Nominal $-\log_{10}(p_{PP})$
Empirical $-\log_{10}(q_{PP})$
0
1
2
3
4
5
6
7
All SNPs
$p_{NEUR} < 10^{-1}$
$p_{NEUR} < 10^{-2}$
$p_{NEUR} < 10^{-3}$
Expected

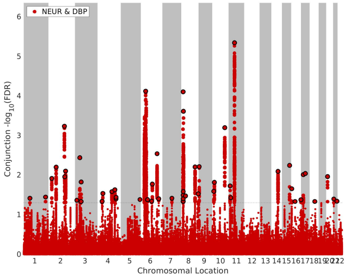
NEUR & DBP
Conjunction -log10(FDR)
Chromosomal Location
0
1
2
3
4
5
6
1
2
3
4
5
6
7
8
9
10
11
12
13
14
15
16
17
18
19
20
21
22

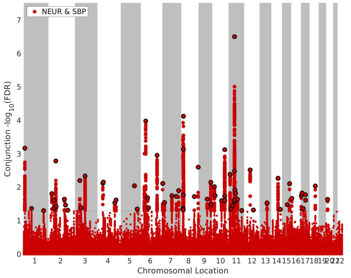
NEUR & SBP
Conjunction -log10(FDR)
0
1
2
3
4
5
6
7
1
2
3
4
5
6
7
8
9
10
11
12
13
14
15
16
17
18
19
20
21
22
Chromosomal Location

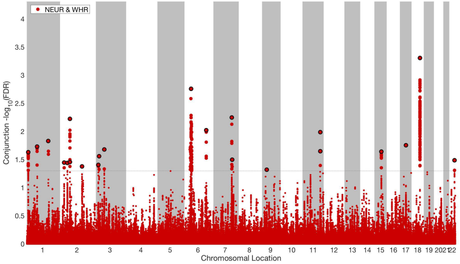

NEUR & WHR
Conjunction -log10(FDR)
0
0.5
1
1.5
2
2.5
3
3.5
4
1
2
3
4
5
6
7
8
9
10
11
12
13
14
15
16
17
18
19
20
21
22
Chromosomal Location

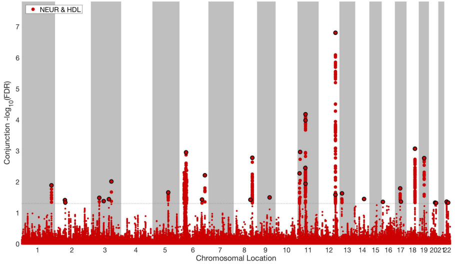
NEUR & HDL
Conjunction -log10(FDR)
0
1
2
3
4
5
6
7
1
2
3
4
5
6
7
8
9
10
11
12
13
14
15
16
17
18
19
20
21
22
Chromosomal Location

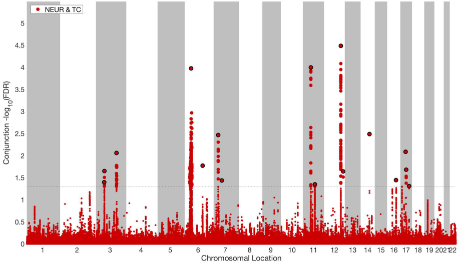

NEUR & TC
Conjunction -log10(FDR)
0
0.5
1
1.5
2
2.5
3
3.5
4
4.5
5
1
2
3
4
5
6
7
8
9
10
11
12
13
14
15
16
17
18
19
20
21
22
Chromosomal Location

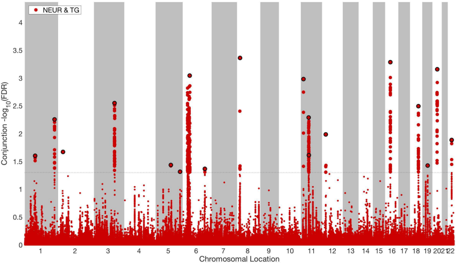
NEUR & TG
Conjunction -log10(FDR)
4
3.5
3
2.5
2
1.5
1
0.5
0
1
2
3
4
5
6
7
8
9
10
11
12
13
14
15
16
17
18
19
20
21
22
Chromosomal Location

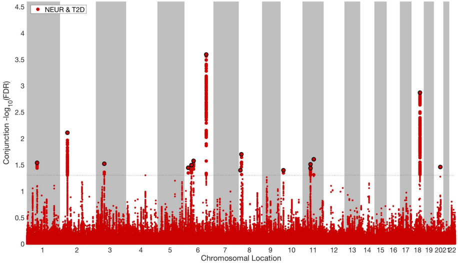
NEUR & T2D
Conjunction -log10(FDR)
Chromosomal Location
0
0.5
1
1.5
2
2.5
3
3.5
4
4.5
1
2
3
4
5
6
7
8
9
10
11
12
13
14
15
16
17
18
19
20
21
22

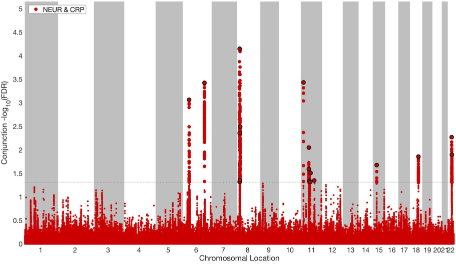

NEUR & CRP
Conjunction -log10(FDR)
0
0.5
1
1.5
2
2.5
3
3.5
4
4.5
5
1
2
3
4
5
6
7
8
9
10
11
12
13
14
15
16
17
18
19
20
21
22
Chromosomal Location

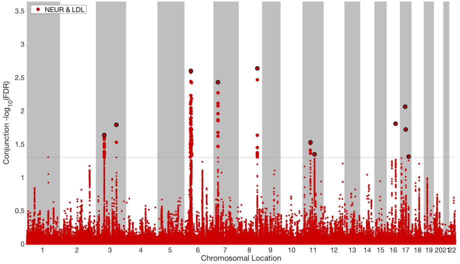

NEUR & LDL
Conjunction -log10(FDR)
0
0.5
1
1.5
2
2.5
3
3.5
1
2
3
4
5
6
7
8
9
10
11
12
13
14
15
16
17
18
19
2021
22
Chromosomal Location

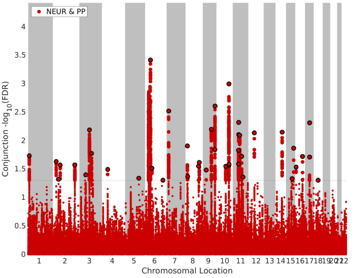

NEUR & PP
Conjunction -log10(FDR)
Chromosomal Location
0
0.5
1
1.5
2
2.5
3
3.5
4
1
2
3
4
5
6
7
8
9
10
11
12
13
14
15
16
17
18
19
20
21
22

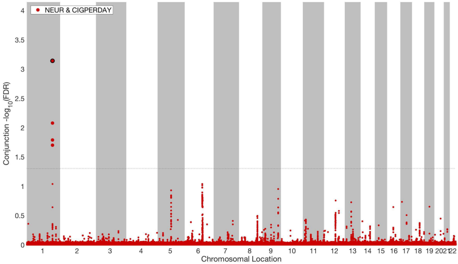
NEUR & CIGPERDAY
Conjunction -log10(FDR)
Chromosomal Location
0
0.5
1
1.5
2
2.5
3
3.5
4
1
2
3
4
5
6
7
8
9
10
11
12
13
14
15
16
17
18
19
20
21
22
